# PLNMFG: Pseudo-label guided non-negative matrix factorization model with graph constraint for single-cell multi-omics data clustering

**DOI:** 10.1371/journal.pcbi.1013375

**Published:** 2025-08-18

**Authors:** Hui Yuan, Mingzhu Liu, Yushan Qiu, Wai-Ki Ching, Quan Zou

**Affiliations:** 1 School of Mathematical Sciences, Shenzhen University, Shenzhen, China; 2 Institute for Advanced Study, Shenzhen University, Shenzhen, China; 3 Department of Mathematics, The University of Hong Kong, Hong Kong, China; 4 Institute of Fundamental and Frontier Sciences, University of Electronic Science and Technology of China, Chengdu, China; University of Rochester Medical Center, UNITED STATES OF AMERICA

## Abstract

The development of single-cell multi-omics sequencing technologies has enabled the simultaneous analysis of multi-omics data within the same cell. Accurate clustering of these cells is crucial for downstream analyses of complex biological functions. Despite significant advances in multi-omics integration approaches, current methodologies exhibit two major limitations. First, they inadequately incorporate prior biological knowledge from various omic layers. Second, these methods often conduct independent dimensionality reduction on individual omic datasets, thereby failing to capture the intrinsic complementary information and potentially overlooking crucial cross-platform interactions. Motivated by these, this study investigates a non-negative matrix factorization model called PLNMFG, which integrates the unified latent representation learning that retains the features between and within omics and the cluster structure learning that retains the intrinsic structure of the data into one joint framework. Specially, PLNMFG performs adaptive imputation to handle dropout events and uses prior pseudo-labels as constraints during the process of collective non-negative matrix factorization, as a result, a more robust latent representation that preserves the double similarity information is obtained. Graph Laplacian constraint is applied during clustering which further preserves structure characteristic of multi-omics data. In addition, the weight of each omic is adaptively learned based on the omic contribution. A series of experiments on 8 benchmark datasets show that our model performs well in terms of clustering accuracy and computational efficiency.

## Author summary

With the rapid advancement of biotechnology, we can obtain single-cell multi-omics data including genomics, transcriptomics, epigenomics, proteomics, and metabolomics. Single-cell clustering based on these omics data can help to understand the cell heterogeneity, enabling more precise analysis of the human body at the individual cell level, thereby advancing comprehension of human systems. However, because of the high-dimensional and sparse characteristics of single-cell multi-omics data, the clustering performance is generally poor. In this paper, pseudo-label guided non-negative matrix factorization model with graph constraint (PLNMFG) is proposed for analyzing single-cell multi-omics data. It is the first time to integrate pseudo-labels, imputation and clustering based on non-negative matrix factorization and it can be conducted the different task simultaneously in a unified manner. PLNMFG combines imputation techniques with non-negative matrix factorization to further enhance clustering accuracy. It applies an adaptive omics weighting strategy to match the importance of each omic layer, giving more influence to critical omics during the clustering process. And PLNMFG employs collective matrix decomposition method based on pseudo-labeling constraints and thus avoids the traditional computationally intensive feature decomposition and similarity graph construction. Furthermore, PLNMFG applies manifold constraints in the clustering process to further preserve the data structure, it simultaneously learns the latent representation and clustering structure in the same framework, making the latent representation more suitable for clustering. Experimental results on eight different datasets indicate that PLNMFG method achieves outstanding clustering performance, fully validating its effectiveness and generalization ability.

## 1. Introduction

Cells are fundamental units of the human body. In fact, cells within the same tissue, organ, or cell type may contribute differently to physiological or pathological processes. Understanding heterogeneity at the single-cell level is essential for gaining insights into developmental biology, disease mechanisms, and therapeutic strategies. With the rapid advancement of biotechnology, researchers are now able to obtain single-cell multi-omics data including genomics, transcriptomics, epigenomics, proteomics, and metabolomics [1,2]. Single-cell clustering based on these omics data provides a detailed understanding of heterogeneity, enabling more precise analysis of the human body at the individual cell level, thereby advancing comprehension of human systems.

However, the different data characteristics of individual omic create challenges for cross-omics clustering. For example, Antibody-Derived Tags (ADT) histology data can sequence cell surface proteins at a low loss rate, but are limited by technology to analyze only a few hundred proteins, and thus failing to capture rare or minor cell types effectively. In contrast, the whole transcriptome of mRNA data can capture a wide range of cell types, but its corresponding scRNA-seq data suffers from significant dropout, with more than 80% or even 90% of zero entries. These zeros can be categorized into true zeros (the certain genes that are not expressed in individual cell) and false zeros (the uncertain genes were failure to be detected during the sequencing process). False zeros caused by dropout cannot be distinguished from true zeros, thus affecting the performance of downstream analysis. Thus, imputation the scRNA-seq data is an urgent need when conducting clustering.

In addition to the inherent features of the data, the similarity between the data is not accurate due to the noisy effects of high-dimensionality and heterogeneity [6]. Thus, dimensionality reduction is crucial. Methods like Seurat [7] and TSCAN [8] reduce high-dimensional data using Principal Component Analysis (PCA), projecting features into lower-dimensional space to identify those with maximum variance. Seurat uses Shared Nearest Neighbor (SNN) graph and Louvain’s algorithm for clustering, while TSCAN is based on Minimum Spanning Tree (MST) combined with Gaussian mixture model. However, Seurat struggles with sparse data, and TSCAN suffers from significant computational complexity when scaling to large datasets due to inherent algorithmic limitations.

Non-negative matrix factorization (NMF) has been extensively studied for its wide applications in dimensionality reduction [9], recommender systems [10], and bioinformatics. In addition, researchers has developed multiple methods based on NMF for clustering. For instance, WSNMF [11] incorporates attribute similarity and graph regularization for attributed graph clustering, while AGNMF-AN [12] adaptively learns affinity matrices and applies robust $\ell_{2,1}$ norm constraints to handle noise and outliers. In a broader context, recent surveys [13] have highlighted the integration of NMF and spectral clustering with graph structure learning, emphasizing its relevance in high-dimensional and multi-view data settings. Multi-view NMF (multiNMF) [14] proposed a joint clustering algorithm based on multi-omics NMF by adding regularization constraints to the coefficient matrix, thereby creating a unified latent representation. However, multiNMF only considers inter-omics similarity and ignores intra-omic similarity. Multi-Manifold Regularized NMF (MMNMF) [15] combines multiNMF with NMF to preserve the local structure information of the original data within the coefficient matrices. NMF-CC method further extends the application of NMF [16] by introducing orthogonality constraints on both the original matrix and coefficient matrix which improves the learned representations for multi-omics clustering. Multi-view clustering based on non-negative matrix factorization and pairwise measurements (MPMNMFs) [17] integrates pairwise co-regularization and manifold regularization with NMF. The scMNMF [18] performs dimensionality reduction and cell clustering simultaneously via NMF, however, it does not consider omic contribution. PLCMF utilizes similarity of intra-omic knowledge to guide potential extraction but fails to fully consider the specific data features of each omic layer [19].

In contrast, our proposed PLNMFG model introduces pseudo-label guided regularization, enabling the integration of external prior knowledge without demanding explicit label annotation. This strategy not only strengthens cluster consistency but also avoids the computational overhead associated with constructing full similarity graphs. Furthermore, our method allows flexible omic-specific weighting, enabling better adaptation to data heterogeneity across modalities. These innovations distinguish PLNMFG both in its theoretical formulation and in its practical efficiency compared to existing methods.

In addition, deep learning methods for single-cell multi-omics data clustering have been developed in recent years. For instance, MoClust [3] handles each omic separately using multiple autoencoders, while TotalVI [4] uses a Bayesian framework with a single autoencoder for joint modeling, which is computationally intensive and sensitive to data quality. scMVP [5] integrates scRNA-seq and scATAC-seq using a multi-view variational autoencoder with attention and Gaussian mixture priors, but it focuses on paired data and requires complex training, limiting its generalizability.

**Fig 1:**
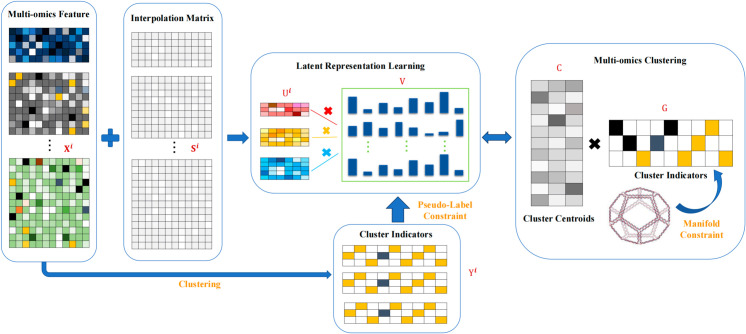
The overall framework of PLNMFG. The model first applies dropout-aware imputation to recover false zeros in each omic via sparse correction. Pseudo-labels are then generated by applying *k*-means clustering independently to each omic, guiding the joint matrix factorization. Multi-omics data are simultaneously factorized with pseudo-label regularization to obtain a unified latent representation. Adaptive modality weighting adjusts the contribution of each omic via a power-law weighting scheme. Finally, manifold regularization is imposed on the cluster indicator matrix to preserve both global cross-omic consistency and local sample geometry.

Overall, existing single-cell multi-omics clustering methods may overlook the prior knowledge of each omic and lack effective techniques to handle data dropout events. Additionally, they perform dimensionality reduction separately for each omics layer, resulting in the loss of complementary information. To address these challenges, we propose a novel multi-omics matrix factorization clustering method PLNMFG. The proposed PLNMFG adaptively imputes each omic dataset and then applies non-negative matrix factorization method with pseudo-label constraints to learn a unified latent representation. The pseudo-labels of PLNMFG are derived from the clustering results of each individual omic, which effectively utilizes prior knowledge and preserves the intra-omic characteristic. The unified latent representation obtained from the collective non-negative matrix factorization retains the complementary information between omics. As a result, both intra-omics and inter-omics similarities are preserved. Next, the PLNMFG performs clustering based on the unified latent representation and imposes a graph Laplacian constraint on the clustering results. By introducing the graph Laplacian regularization term, the clustering algorithm significantly enhances its ability to capture the intrinsic manifold structure of the data. Finally, PLNMFG integrates imputed pseudo-label constrained unified latent representation learning and manifold structure-preserving cluster structure learning into one unified framework. This integration ensures that each step of the algorithm strengthens the results and further enhancing the clustering performance. Specific flowchart of PLNMFG is illustrated in Fig 1.

## 2. Materials and methods

### 2.1. Notations and problem formulation

Let $𝒪=\{o_{j}{\}}_{j=1}^{n}$ be the multi-omics dataset, where *v* is the number of omics and *n* is the number of samples. Feature vector ${𝐱}_{j}^{i}$ is the *j*-th column vector of the *i*-th data matrix ${𝐗}^{i}=[{𝐱}_{1}^{i},{𝐱}_{2}^{i},\ldots ,{𝐱}_{n}^{i}]\in {\mathbb{R}}^{d_{i}\times n}$, where *d*_*i*_ is the feature dimension of the *i*-th omic.

Given *c* clusters, the goal of the PLNMFG algorithm is to assign the *n* data points to *c* groups, ensuring that data points with similar characteristics are placed in the same group. Table 1 summarizes the main notations and their descriptions used in this paper.

**Table 1:** Notations and descriptions.

Notation	Description	Size
${𝐗}^{i}$	Data matrix of the *i*-th omic	$d_{i}\times n$
${𝐔}^{i}$	Latent factor matrix of the *i*-th omic	$d_{i}\times k$
${𝐐}^{i}$	Linear projection matrix of the *i*-th omic	c×k
${𝐘}^{i}$	Pseudo-label matrix of the *i*-th omic	c×n
**V**	Unified coefficient matrix	k×n
**C**	Clustering centroid matrix	k×c
**G**	Indicator matrix	c×n
${𝐒}^{i}$	Imputation matrix of the *i*-th omic	$d_{i}\times n$
*d*_*i*_	Dimensionality of the *i*-th omic	${\mathbb{N}}_{+}$
*n*	Number of samples	
*k*	Dimensionality of the latent space	
*v*	Number of omics	
*c*	Number of clusters	

### 2.2. Unified latent representations learning

#### 2.2.1. Collective matrix decomposition.

To promote clustering results, PLNMFG projects the features of different omics into the same low-dimensional latent space. The following formula achieves this goal by minimizing the Frobenius norm between data matrix of the *i*-th omic ${𝐗}^{i}$ and its corresponding low-rank decomposition ${𝐔}^{i}𝐕$:

$$\min\sum_{i=1}^{v}(\alpha_{i}{)}^{\gamma }\|{𝐗}^{i}-{𝐔}^{i}𝐕{\|}_{F}^{2},\text{s.t.}\sum_{i=1}^{v}\alpha_{i}=1,\alpha_{i}>0.$$ (1)

Here ${𝐔}^{i}=[{𝐮}_{1}^{i},{𝐮}_{2}^{i},\ldots ,{𝐮}_{k}^{i}]\in {\mathbb{R}}^{d_{i}\times k}$ is the latent factor vectors for the *i*-th omic, ${𝐔}^{i}$ projecting data features of ${𝐗}^{i}$ into a low-dimensional space to obtain a unified coefficient vector **V** that represents the combined feature space of all omics. Here *k* denotes the number of latent factors, $\alpha =[\alpha_{1},\alpha_{2},\ldots ,\alpha_{n}]\in {\mathbb{R}}^{v}$ is a non-negative normalization weight vector to balance the contribution of each omics modality, and γ>1 is a parameter controlling distribution.

Through collective matrix factorization, each feature vector ${𝐱}_{j}^{i}$ in the *i*-th omics is approximated as a linear combination of latent factor ${𝐔}^{i}$ weighted by the corresponding coefficient $v_{j}$. Therefore, ${𝐔}^{i}$ can be viewed as forming the basis vectors of the latent space hidden in the *i*-th omic, and **V** represents the unified latent representations across all omics.

#### 2.2.2. Imputation technique.

To recover the “false zero” caused by the dropout events, we define the matrix **S** and establish a predefined threshold [20,21]. When the probability of entry ${𝐗}_{ij}$ in the initial matrix **X** exceeds this threshold, we designate the corresponding entry as a “false zero”. In this case, ${𝐒}_{ij}>0$; otherwise, ${𝐒}_{ij}=0$. The reconstructed matrix for the *i*-th omic can be expressed as ${𝐗}^{i}\;+\;{𝐒}^{i}$ and we perform collective matrix factorization on all imputation matrices:

$$\min\sum_{i=1}^{v}(\alpha_{i}{)}^{\gamma }\left\{\|{𝐗}^{i}+{𝐒}^{i}-{𝐔}^{i}𝐕{\|}_{F}^{2}+\eta \sum_{j=1}^{n}u_{j}\|{𝐒}_{j}^{i}{\|}_{1}\right\}.$$ (2)

The parameter *u*_*j*_ of regularization term $\eta \sum_{j=1}^{n}u_{j}||{𝐒}_{j}^{i}{\left|\right|}_{1}$ represents the sequencing depth of different columns. This is because different cell data have different sequencing depths, as deeply sequenced cells are expected to have lower dropout rates when compared to shallowly sequenced ones. Regarding the imputation matrix ${𝐒}^{i}$, since zero entries encompass both dropout events and true zero expressions, and the exact locations of all dropout events remain unknown, we can reasonably assume that ${𝐒}^{i}$ is a sparse matrix. Consequently, we impose the *L*_1_-norm constraints on ${𝐒}^{i}$.

#### 2.2.3. Pseudo-label constraint.

Each omic contains valuable clustering information, we derive pseudo-labels by clustering on each omic separately to effectively leverage this kind of prior information. Given that *k*-means clustering is widely used due to its simplicity and low computational cost, we use *k*-means as the basic strategy for generating pseudo-labels. The process of generating pseudo-labels is a pre-processing step in the PLNMFG method. We apply *k*-means clustering to the feature vectors of each omic data ${𝐗}^{i}(i=1,2,\ldots ,v)$ into *c* groups, resulting in *c* different clusters for the *i*-th omic: $𝒞=\{C_{1}^{i},C_{2}^{i},\ldots ,C_{c}^{i}\}$. The binary matrix ${𝐘}^{i}=[y_{1}^{i},y_{2}^{i},\ldots ,y_{n}^{i}]\in \{0,1{\}}^{c\times n}$ is the cluster indicator matrix for the *i*-th omic, ${𝐘}^{i}(t,m)=1$ if data point $x_{m}^{i}$ belongs to the *t*-th cluster, otherwise ${𝐘}^{i}(t,m)=0$. Cluster indicator matrix ${𝐘}^{i}$ serves as the pseudo-labels for each omic modality. To capture intra-omics similarity, we introduce a pseudo-label constraint:

$$\sum_{i=1}^{v}\|{𝐘}^{i}-{𝐐}^{i}𝐕{\|}_{F}^{2}.$$ (3)

Here ${𝐐}^{i}\in {\mathbb{R}}^{c\times k}$ is the linear projection matrix that maps the unified latent representation **V** into the binary pseudo-label space ${𝐘}^{i}$ for the *i*-th omics. The pseudo-label constraint ensures that similar feature data share same pseudo-label and preserves the similarity structure within each omics modality through the unified latent representation **V**.

The formula is then shown as follows:

$$\min\sum_{i=1}^{v}(\alpha_{i}{)}^{\gamma }\{\|{𝐗}^{i}+{𝐒}^{i}-{𝐔}^{i}𝐕{\|}_{F}^{2}+\eta \sum_{j=1}^{n}u_{j}\|{𝐒}_{j}^{i}{\|}_{1}+\delta \|{𝐘}^{i}-{𝐐}^{i}𝐕{\|}_{F}^{2}\}\;\text{s.t.}\;\sum_{i=1}^{v}\alpha_{i}=1,\;\alpha_{i}>0.$$ (4)

Here δ>0 is a parameter controlling the significance of the pseudo-label constraint on the entire objective function.

### 2.3. Cluster structure learning

#### 2.3.1. Indicator matrix.

To derive clustering indicators from the learned latent representation **V**, we propose a clustering structure learning formulation:

$$\min\|𝐕-𝐂𝐆{\|}_{F}^{2}.$$ (5)

The clustering structure learning formulation (5) decomposes the previously learned unified latent representation **V** into a clustering centroid matrix **C** and an indicator matrix **G**.

#### 2.3.2. Manifold regularization constraint.

To better incorporate the geometric structure of the original data, we impose graph Laplacian constraints on **G**. In [22], it is proved that the local topology structure preservation can be formulated as trace optimization, i.e.,


$$\frac{1}{2}\sum_{i,j}w_{ij}||g_{.i}-g_{.j}|{|}^{2}=\mathrm{Tr}(GLG^{T}).$$

And the specific steps of getting the graph Laplacian matrix **L** are as follows. Firstly, construction of the graph adjacency matrix *A*: Construct a matrix such that *A*_*ij*_ = 1 indicates an edge between nodes *i* and *j*, and *A*_*ij*_ = 0 indicates no connection. Then, construction of the graph degree matrix D: Construct a diagonal matrix where *D*_*ii*_ represents the degree of node *i*, which is the number of edges connected to node *i*. Finally, computing the graph Laplacian matrix *L*: Apply the formula L=D−A.

By applying manifold regularization constraint on **G** through $\mathrm{Tr}({GLG}^{T})$, we obtain the clustering structure learning formulation:

$$\beta \|𝐕-𝐂𝐆{\|}_{F}^{2}+Tr(𝐆𝐋{𝐆}^{T})$$ (6)

The constraint ensures that the group indicator matrix obtained by PLNMFG preserves the geometric structure of the omics data in the original space. The parameters *β* and *ε* are non-negative weight parameters that control the clustering structure learning term and the manifold structure regularization term.

#### 2.3.3. Overall objective function.

Therefore, the objective function of PLNMFG is defined by combining the unified latent representations learning (4) and the clustering structure learning term (6). The objective function is given as follows:

$$\min ℱ({𝐒}^{i},{𝐔}^{i},{𝐐}^{i},𝐕,𝐂,𝐆,\alpha_{i})\;=\sum_{i=1}^{v}(\alpha_{i}{)}^{\gamma }\{\|{𝐗}^{i}+{𝐒}^{i}-{𝐔}^{i}𝐕{\|}_{F}^{2}+\eta \sum_{j=1}^{n}u_{j}\|{𝐒}_{j}^{i}{\|}_{1}+\delta \|{𝐘}^{i}-{𝐐}^{i}𝐕{\|}_{F}^{2}\}\;+\beta \|𝐕-𝐂𝐆{\|}_{F}^{2}+\varepsilon \mathrm{Tr}(𝐆𝐋{𝐆}^{T})\;\text{s.t.}\;\sum_{i=1}^{v}\alpha_{i}=1,\;\alpha_{i}\geq 0.$$ (7)

All parameters-selection details of the PLNMFG model are given in the S1 Text. And the the details of the iterative optimization of parameters is provided in the S2 Text, along with the convergence proof (see details in S5 Text).

## 3. Experimental results and analysis

### 3.1. Datasets

We employed six real-world datasets and two simulated datasets as benchmark datasets for evaluating the performance of PLNMFG method. All datasets are pre-processed, and specific pre-processing steps are provided in S3 Text. Detailed information about these datasets is provided in Table 2.

**Table 2:** Summary of the single-cell multi-omics datasets.

Dataset	Cells	RNA	ADT	ATAC	Types
Spector	3,762	33,538	49	–	16
10X_10K	6,661	33,538	17	–	7
BMNC	30,672	17,009	25	–	27
SMAGE	11,020	36,611	–	20,010	12
Anno	1,182	5,000	10	–	6
Pbmc	7,865	499	13	–	8
Sim1	530	2,000	–	5,000	3
Sim2	1,000	2,000	30	–	8

### 3.2. Comparison of methods

To evaluate the performance of PLNMFG, we selected nine state-of-the-art methods for comparisons, focusing on five aspects: clustering, dimensionality reduction, imputation, pseudo-labels, and matrix factorization. redWe listed information about related methods in Table 3. For clustering performance comparison, we benchmarked PLNMFG against the traditional method Seurat and deep learning clustering algorithms MoClust [3], TotalVI [4], and scMVP [5]. Seurat employs shared nearest neighbor graphs for clustering to preserve structural features; MoClust drives clustering vectors toward orthogonality and simplex through Cauchy-Schwarz divergence; TotalVI, like our method, performs clustering in latent space; and scMVP integrates multi-modal variational inference with contrastive learning to jointly embed cells and genes, enabling accurate and scalable clustering across different omics layers.

**Table 3:** Summary of the comparison methods.

Methods	Language	Link	Principle
MOFA+	R/Python	https://github.com/satijalab/seurat	Probabilistic Factor Analysis
Seurat	R	https://github.com/satijalab/seurat	Graph Theory
Tscan	R	https://github.com/zji90/TSCAN	
MoClust	Python	https://github.com/ddb-qiwang/MoClust	Deep learning
TotalVI	Python	https://github.com/YosefLab/totalVI_reproducibility	
scMVP	Python	https://github.com/bm2-lab/scMVP	
scMNMF	Matlab	https://github.com/yushanqiu/scMNMF	Non-matrix Decomposition
BREM-SC	R	https://github.com/tarot0410/BREMSC	
PLCMF	Matlab	https://github.com/Wangdi-Xidian/PLCMF	

To assess dimensionality reduction performance, we selected both MOFA+ [23] and TSCAN [8] for comparisons, as both methods are capable of processing high-dimensional multi-omics data and extracting low-dimensional representations. MOFA+ is a non-deep probabilistic factor analysis model specifically designed for multi-omics integration. It extracts shared and modality-specific latent factors from multi-view data and enables unsupervised clustering in the latent space, providing a robust baseline for evaluating cross-modal structure capture. TSCAN, on the other hand, integrates dimensionality reduction with trajectory inference, making it suitable for characterizing cellular dynamics in a reduced space.

Given that our method is based on the non-negative matrix factorization (NMF) framework, we also included three representative NMF-based methods for comparisons: scMNMF [18], PLNMF [19], and BREM-SC [24]. BREM-SC incorporates multiple prior knowledge sources, performs decomposition based on Bayesian sparse matrices, automatically learns weights for different data modalities, and effectively handles dropout events. The newly proposed scMNMF simultaneously performs imputation and graph clustering under the NMF framework. PLCMF represents a collaborative clustering method under pseudo-label constraints.

The main difference between PLCMF and PLNMFG is that PLCMF performs collective matrix decomposition on preprocessed multi-omics date, while PLNMFG extracts unified latent features from imputation matrices. Additionally, PLNMFG imposes graph Laplacian constraints during the learning of the cluster structure, further enhancing its ability to capture intrinsic sample relationships.

### 3.3. Clustering performance

**Fig 2:**
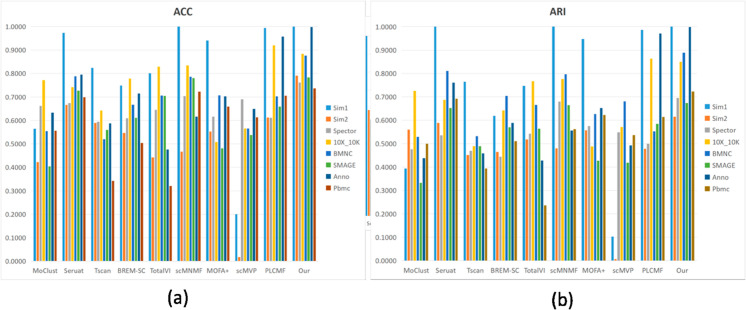
Clustering performance of different methods on different datasets. (a) Performance measured by ACC. (b) Performance measured by ARI.

To evaluate the clustering performance, four metric including Accuracy (ACC), Adjusted Rand Index (ARI), Adjusted Mutual Information (AMI), Normalized Mutual Information (NMI) are employed, and the definition of these metric are given in the S4 Text. The clustering results of PLNMFG and the competing methods are evaluated using ACC and ARI on eight different datasets which are shown in Fig 2. PLNMFG demonstrated superior clustering performance across almost all experimental datasets. Additional performance metrics AMI and NMI are provided in S1 Fig, the results also confirm the superiority of our proposed method.

#### 3.3.1. Performance analysis of PLNMFG on different dataset scales.

We analyzed the performance of the methods across different datasets scales. PLNMFG demonstrated outstanding accuracy on small- to large-scale datasets. Notably, on the Sim1 (530 cells) and Anno (1182 cells) datasets, PLNMFG achieved near-perfect performance, with ACC and ARI values approaching 1. For the large-scale BMNC dataset (30,672 cells), PLNMFG attained an impressive ACC of 0.8768, significantly surpassing other benchmark methods, which indicates the methods strong generalizability to large-scale settings. Furthermore, the method maintained competitive performance on the Spector dataset (16 cell types), underscoring its robustness in handling high cell-type heterogeneity. However, MOFA+, a multi-modal factor analysis model specifically designed for integrating diverse data modalities, exhibited less stable performance across datasets. While MOFA+ is particularly effective when applied to datasets with well-defined multi-modal structuressuch as Sim1 and Sim2, its performance tends to degrade on large, sparse, and noisy datasets such as 10X_10K and PBMC. This decline can be attributed to the models complexity and its reliance on optimal hyperparameter tuning, which may lead to underfitting or overfitting if not properly addressed. Moreover, MOFA+ exhibits limited robustness in handling the challenges posed by high-dimensional sparsity and heterogeneity commonly found in large-scale single-cell datasets. These observations suggest that MOFA+ is more suitable for moderate-sized datasets with clear multi-modal signals, whereas PLNMFG offers broader applicability and consistent performance across diverse dataset scales and complexities.

#### 3.3.2. Comparison with PCA-based methods.

**Fig 3:**
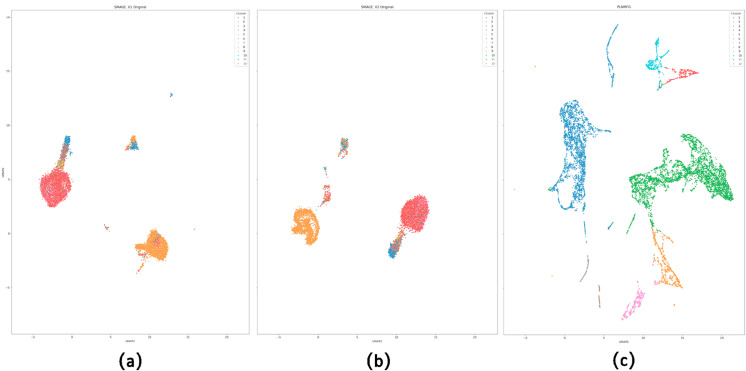
UMAP visualization on the SMAGE dataset. (a) RNA data of SMAGE. (b) ADT data of SMAGE. (c) unified latent represent learned from SMAGE by PLNMFG.

We compare PLNMFG with Tscan and Seurat, which depend on traditional PCA dimensionality reduction. Tscan performed poorly on datasets such as 10X_10K and PBMC, probably because the PCA fails to capture subtle differences between cells when dealing with highly complex and heterogeneous datasets, Tscan performs relatively well on the simulated dataset Sim1 with only three cell types, further confirming our idea. Seurat achieved strong performance on simple datasets (Sim1), but exhibited low ACC values on datasets like 10X_10K, BMNC, and Anno, revealing the instability of its clustering performance. The possible reason is that PCA dimensionality reduction makes Seurat sensitive to outliers such as technical noise and batch effects, and the parameter settings of the principal components (PCs) have a significant impact on the results. In contrast, the collective non-negative matrix factorization approach employed by PLNMFG effectively reduces the dimensionality of multi-omics data. As illustrated in Fig 3, the UMAP visualization of the SMAGE dataset (11,020 cells, 12 cell types) demonstrates that PLNMFG achieves a clearer data distribution and significantly enhances cluster separability. The third panel of Fig 3 shows that cells previously intertwined in the original data are separated after dimensionality reduction. The Umap visualization for other datasets can be found in the S2 Fig.

#### 3.3.3. Comparison with deep learning methods.

The deep learning method MoClust demonstrated moderate performance across most datasets, with particularly poor results on SMAGE and Sim1. This limitation can be attributed to the instability of MoClust’s Gaussian mixture model estimation when applied to high-dimensional and sparse scRNA-seq data, where the majority of gene expression values are zero. TotalVI performs averagely in RNA and ATAC omics (Sim1 and SMAGE), which may be because TotalVI assumes a certain correlation between different omics and integrates them through a joint model. If the biological features or technical characteristics of the two types of data differsubstantially, it is easy to be constricted when integrating heterogeneous data and leading to integration failure. scMVP shows relatively poor performance on several datasets, especially Sim1, Sim2, and Anno, likely due to its sensitivity to data sparsity and noise in the contrastive learning framework. In contrast, PLNMFG consistently outperforms scMVP across all metrics and datasets, demonstrating stronger robustness on heterogeneous real-world data such as Anno and PBMC. This highlights the effectiveness of our pseudo-label guidance and omic-specific regularization mechanisms.

#### 3.3.4. Comparison with NMF-based methods.

Among NMF-based methods, BREM-SC showed average performance on most datasets. This could be due to its Bayesian framework’s heavy reliance on prior distribution assumptions. If these assumptions deviate significantly from the actual data distribution, the model’s results may not accurately capture true biological features. Additionally, BREM-SC does not explicitly model the phenomenon of zero inflation, which limits its ability to handle dropout events effectively. scMNMF exhibited relatively weaker performance on large-scale datasets such as Spector and BMNC. This may be owing to its feature extraction and dimensionality reduction processes rely on a shared basis matrix *W*. When handling large-scale data, the dimensions of *W* may be insufficient to capture all biologically relevant features, leading to information loss. Furthermore, scMNMF does not explicitly assign weights to different omics modalities. Lacking of weighting will result in imbalanced information integration, thereby affecting dimensionality reduction and clustering outcomes. In contrast, PLNMFG incorporates iterative weighting for each omics modality, enabling it to flexibly adjust based on the contribution of each modality.

The clustering performance of PLCMF declined on datasets with high heterogeneity and complex cellular structures (Sim2, SMAGE, and Spector). This may be due to the loss of structural information in cellular data when the number of cell types is large. In the comparison, the proposed PLNMFG method incorporates manifold structure constraints, which effectively mitigate this issue by preserving the structural information of the data.

**Fig 4:**
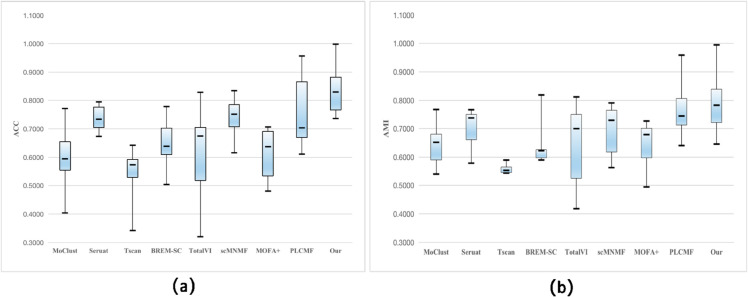
Boxplot of PLNMFG and other eight state-of-the-art algorithms measured by (a) ACC and (b) AMI on six real datasets.

To further validate the feasibility of our model on six real-world datasets, we generated Boxplots based on clustering results to demonstrate the overall clustering performance of PLNMFG compared to other methods. For each dataset, we computed the lower quartile, maximum, minimum, median, and upper quartile of the clustering performance metrics for each method. These values were then visualized as Boxplots to facilitate the comparison of the methods’ overall performance. The height of the boxes represents the Inter-Quartile Range (IQR). Smaller IQRs indicate concentrated and stable clustering results, while larger IQRs suggest instability, implying that the method may be unreliable. The ACC and AMI results are shown in Fig 4, and the results for ARI and NMI are presented in S3 Fig. The plots reveal that PLNMFG consistently yields relatively concentrated performance across all datasets, with an average performance significantly better than other tested methods.

In conclusion, the PLNMFG method effectively integrates information from different data sources through the combination of pseudo-label constraints and collective matrix factorization, improving clustering accuracy particularly for datasets with high data correlation and complex internal structures. The manifold regularization constraint preserve the geometric structure of the original data at a lower computational cost to enhance the clustering performance. Overall, the proposed PLNMFG method achieves promising clustering performance.

## 4. Ablation analysis

**Fig 5:**
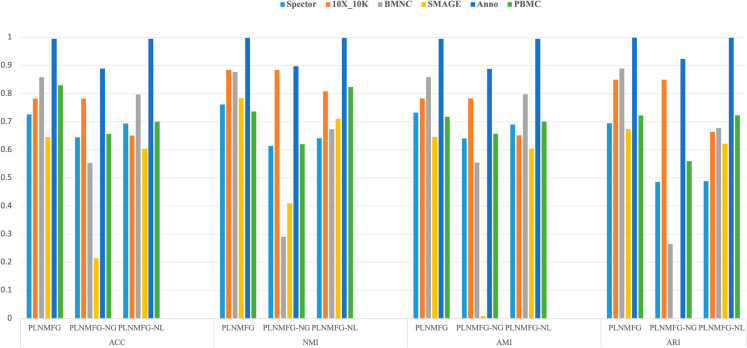
Comparison of ablation experiment results.

PLNMFG_NL denotes the proposed method without the pseudo-label constraint, and PLNMFG_NG refers to the method without the graph Laplacian constraint. The clustering results of PLNMFG_NL and PLNMFG_NG are summarized in Fig 5. We can see that PLNMFG outperforms PLNMFG_NL on most datasets and shows up to 20 improvement on the Specter and 10X_10K datasets, which validates the importance of pseudo-label constraints. Similarly, PLNMFG is superior to PLNMFG_NG on most dataset, suggesting that manifold structure learning effectively enhances clustering results. These findings confirm that both the pseudo-label constraint and the graph Laplacian constraint are crucial for the success of PLNMFG.

## 5. Convergence analysis

**Fig 6:**
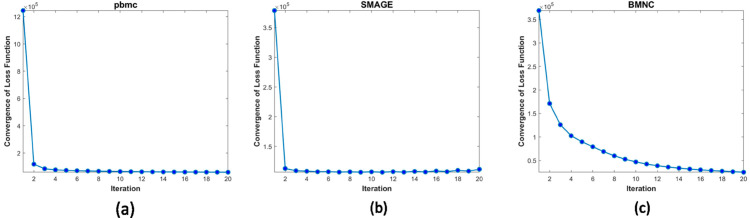
The convergence curves of PLNMFG on different datasets.

As the PLNMFG method is solved through an alternating optimization strategy, we prove that PLNMFG is monotonically non-increasing and the proof can be found in S5 Text. In addition, we also conduct numerical experiments to investigate the convergence speed of PLNMFG. The method demonstrates rapid convergence speed, typically stabilizing within 5 iterations for most datasets (Fig 6(a)-6(b)). For the larger BMNC dataset (Fig 6(c)), convergence is achieved within 15 iterations, demonstrating the method’s scalability to large-scale datasets.

### 5.1. Parameter sensitivity analysis

#### 5.1.1. Analysis of *β*, *δ*, *η* and *γ.*

**Fig 7:**
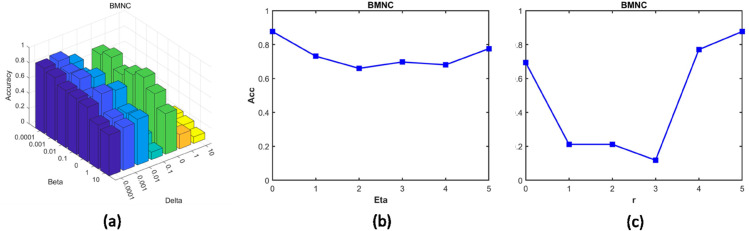
Parameter sensitivity analysis in BMNC dataset. (a) Comprehensive analysis of *β* and *δ*, (b) Line graph with parameter *η* and ACC, (c) Line graph with parameter *γ* and ACC.

We use BMNC dataset as an example to show the impact of the four parameters on clustering performance. Fig 7(a) is the comprehensive analysis of *β* and *δ*, we observed that PLNMFG maintains high and stable cluster performance when β<0.01 and δ<0.1. Fig 7(b) shows that PLNMFG achieves the best clustering performance when *η*=3. Fig 7(c) demonstrates γ=4 should be chosen for the BMNC for highest accuracy.

The records of sensitivity experiments for each parameter of other datasets are shown in S4-S6 Fig. S4 Fig are comprehensive analysis of both *β* and *δ*, while S5 Fig demonstrates the relationship between ACC and the parameter *η*, and S6 Fig demonstrates the relationship between ACC and the parameter *γ*.

Based on the sensitivity curves across multiple datasets, we conclude that PLNMFG generally achieves stable and satisfactory clustering performance when *β* lies within [10^−4^,10] and *δ* within [10^−4^,10]. For the dropout imputation parameter *η*, most datasets achieve optimal or near-optimal accuracy when *η* is selected within [0,5]. Similarly, S6 Fig shows that setting *γ* within [0,5] allows the adaptive weighting mechanism to balance modality contributions effectively while maintaining stable clustering results across datasets. Therefore, in practical applications, we recommend these intervals as empirical search ranges. For small datasets, a grid search within these recommended ranges is feasible, while for larger datasets, random sampling combined with grid search can be employed to efficiently determine suitable parameter values.

#### 5.1.2. Analysis of cluster number *K.*

To investigate the impact of the number of clusters on method performance, we conducted clustering experiments on four datasets using ACC as the evaluation metric. The number of clusters, *K*, was varied from 20 to 200 with an increment of 20. S7 Fig shows ACC distribution for different datasets. We can see that the accuracy for SMAGE remains relatively stable around 0.6, while the one for BMNC fluctuates more, reaching its peak at *K* = 60. For SPECTER, accuracy reaches a local peak at *K* = 80, slightly decreasing at *K* = 100, but overall showing little variation. For PBMC, accuracy is highest at *K* = 100, after which it decreases as *K* increases. This indicates that the biological meaning of the datasets and the number of cell types determine the optimal value of *K*, highlighting the flexibility of our method.

## 6. Discussion

To address the challenges of existing multi-omics clustering methods, we propose the Pseudo-Label Guided Non-negative Matrix Factorization Model with Graph Constraints (PLNMFG) method. This method introduces imputation techniques prior to collective matrix factorization to enhance the robustness of latent representation learning. Furthermore, we incorporate a pseudo-label learning mechanism to preserve intra-omics and inter-omics data structure features based on non-negative matrix factorization. In the clustering process, we apply the graph Laplacian constraints, enabling the PLNMFG method to maintain the manifold structure of the data at a lower computational cost. The proposed method integrates latent representation learning and clustering structure learning into a unified framework, fostering better collaboration between algorithmic sub-steps. Additionally, it adaptively learns the weights of each view during the learning process. Experimental results indicate that, when compare to the existing multi-omics clustering algorithms, the PLNMFG algorithm excels in both accuracy and efficiency across multiple benchmark datasets. Ablation studies further demonstrate that the pseudo-label learning and manifold structure constraints significantly enhance multi-omics clustering performance. Although our method has made progress in handling multi-omics datasets, there is still room for improvement. For example, in this paper, we applied *k*-means to generate pseudo-labels, which are sensitive to random initialization and may affect clustering performance. In our future work, we will explore alternative methods for pseudo-label generation and extend the proposed approach to incomplete multi-view data.

## Supporting information

S1 Fig Clustering performance of different algorithms on eight Datasets. (a) AMI, (b) NMI.
(PDF)

S2 Fig Boxplot of different algorithms on eight datasets. (a) ARI, (b) NMI.
(PDF)

S3 Fig UMAP visualization plots. (a)-BMNC; (b)-10X; (c)-Pbmc; (d)-Anno; (e)-Spector. Each UMAP shows visualization plots of two original omics in the first two panels, and the third panel display visualization plots after processing with PLNMFG.
(PDF)

S4 Fig Comprehensive analysis of *β* and *δ* in (a)-10X, (b)-Pbmc, (c)-SMAGE, (d)-Anno, (c)-Spector. (PDF)

S5 Fig Line graph with ACC and the parameter *η* in (a)-Anno, (b)-Spector, (c)-Pbmc, (d)-SMAGE. (PDF)

S6 Fig Line graph with ACC and the parameter *γ* in (a)-Pbmc, (b)-Spector (c)-SMAGE (d)-Anno (e)-10X. (PDF)

S7 Fig Line graph shows the relationship between clustering accuracy and the number of clusters (K) for PLNMFG on four different datasets. (PDF)

S1 Text Parameter determination. (PDF)

S2 Text Iteration process. (PDF)

S3 Text Data pre-processing. (PDF)

S4 Text Performance evaluation metric. (PDF)

S5 Text Convergence proof (PDF)
