# Supplementary figures and images for: PLNMFG: Pseudo-label guided non-negative matrix factorization model with graph constraint for single-cell multi-omics data clustering
Source: PLoS Comput Biol. 2025 Aug 18;21(8):e1013375. doi: 10.1371/journal.pcbi.1013375 (PMC12416850; doi:10.1371/journal.pcbi.1013375)

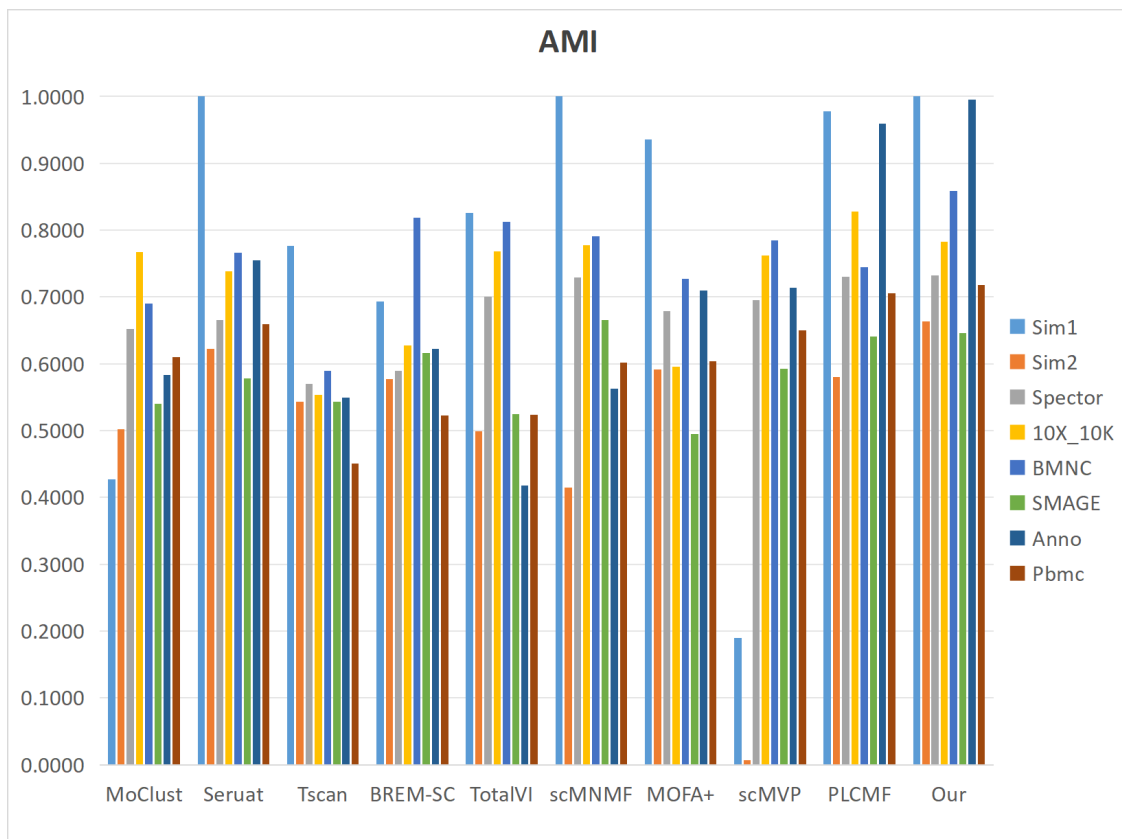

(a)

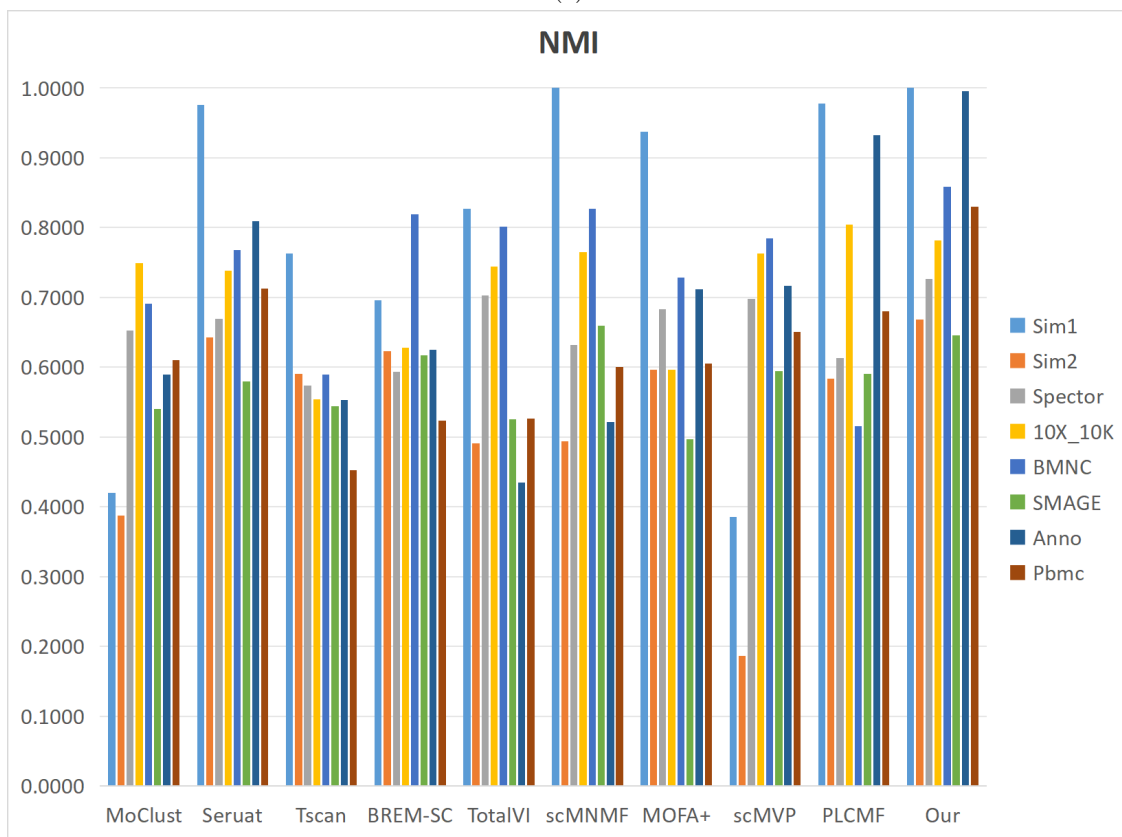

(b)

Clustering performance of different algorithms on eight Datasets. (a) AMI, (b) NMI.

Supplement: S1 Fig — (a) AMI, (b) NMI. (PDF) [file pcbi.1013375.s001.pdf]

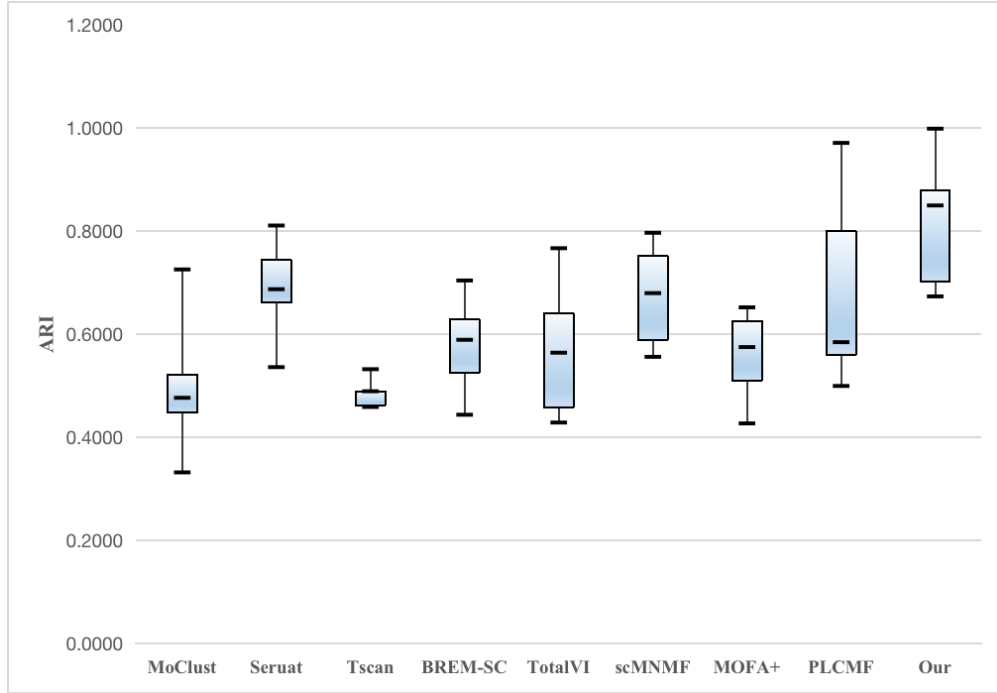

(a)

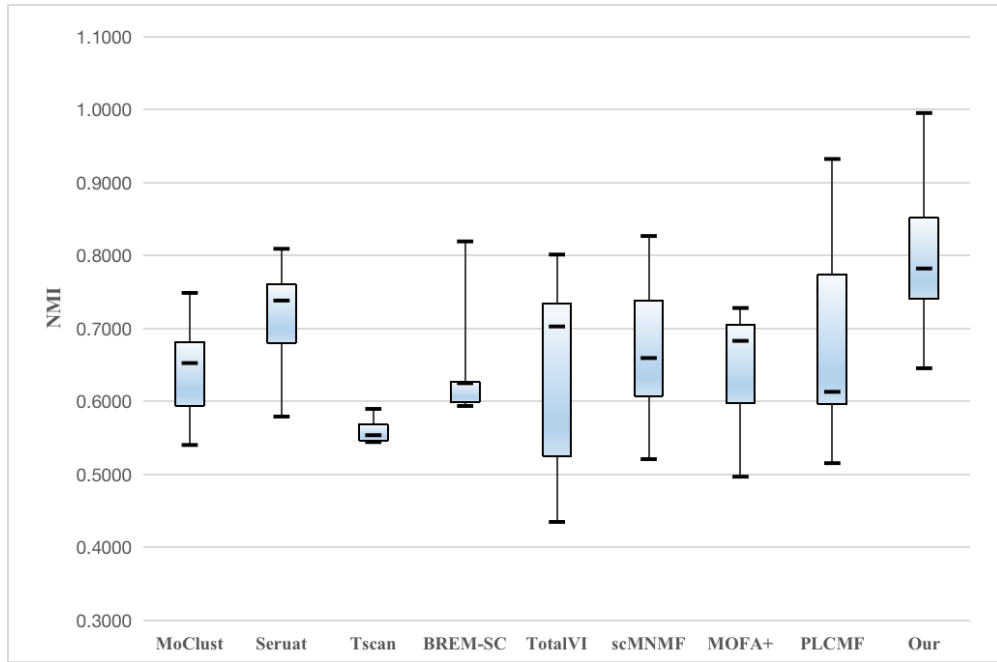

(b)

Boxplot of different algorithms on eight datasets. (a) ARI, (b) NMI.

Supplement: S2 Fig — (a) ARI, (b) NMI. (PDF) [file pcbi.1013375.s002.pdf]

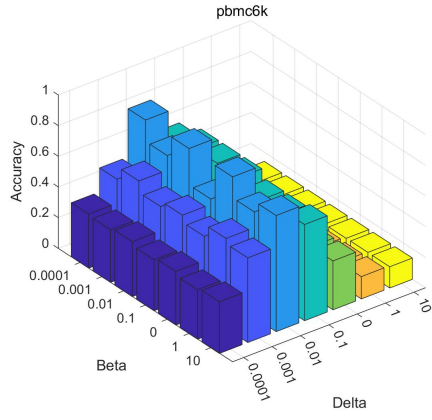

(a)

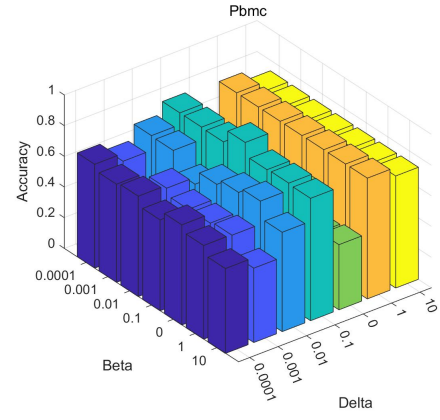

(b)

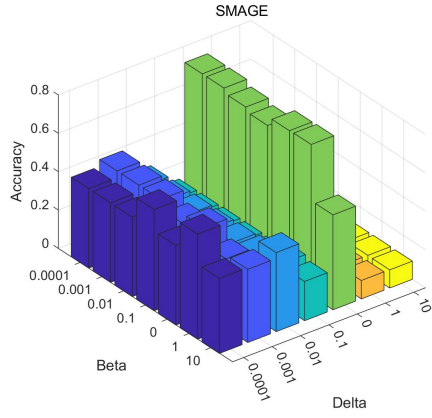

(c)

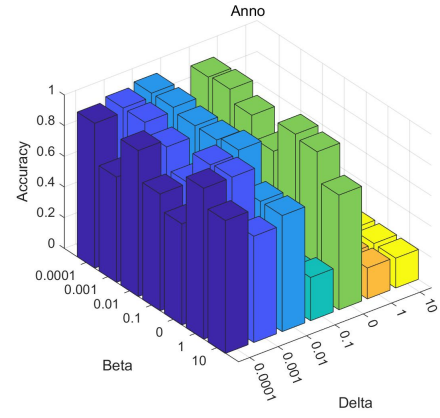

(d)

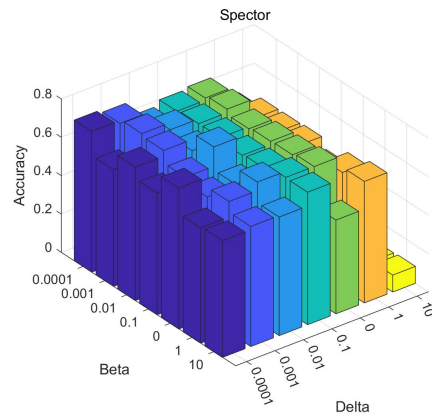

(e)

Comprehensive heatmap of  $\beta$  and  $\delta$  in (a)-10X\_10K, (b)-Pbmc, (c)-SMAGE, (d)-Anno, (e)-Spector.

Supplement: S4 Fig — (PDF) [file pcbi.1013375.s004.pdf]

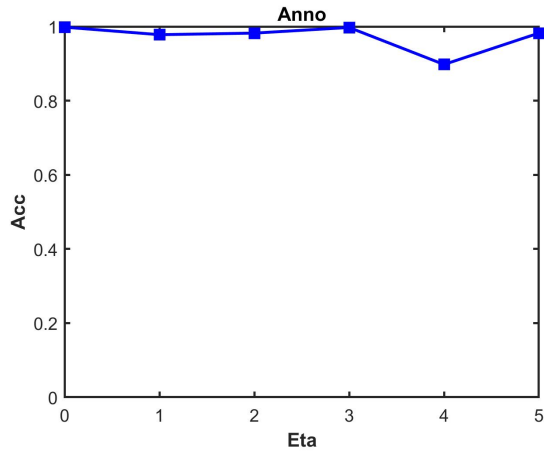

(a)

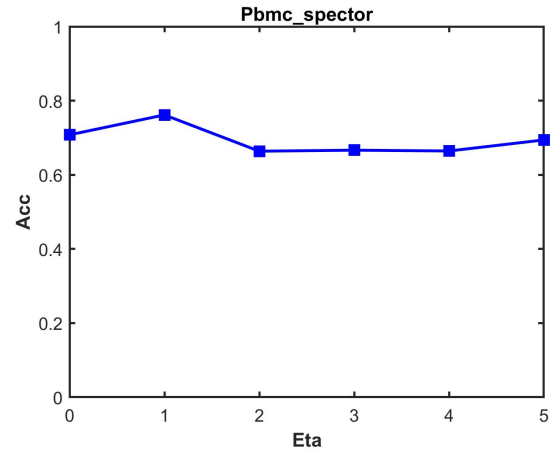

(b)

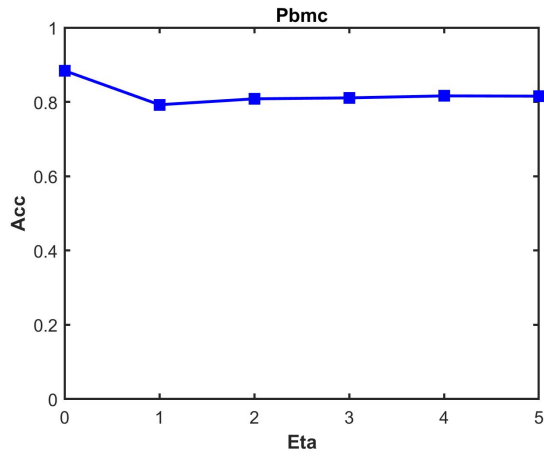

(c)

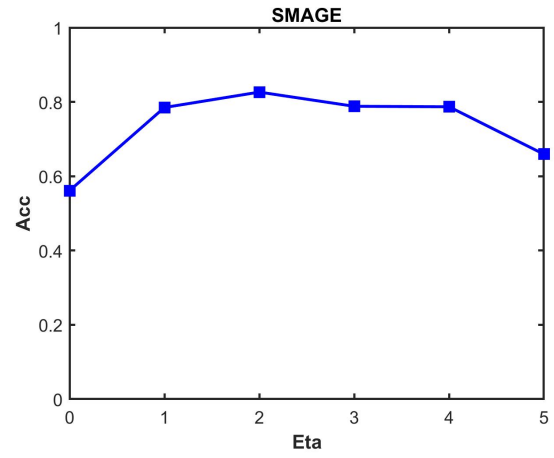

(d)

Line graph with ACC and the parameter  $\eta$  in (a)-Anno, (b)-Spector, (c)-Pbmc, (d)-SMAGE

Supplement: S5 Fig — (PDF) [file pcbi.1013375.s005.pdf]

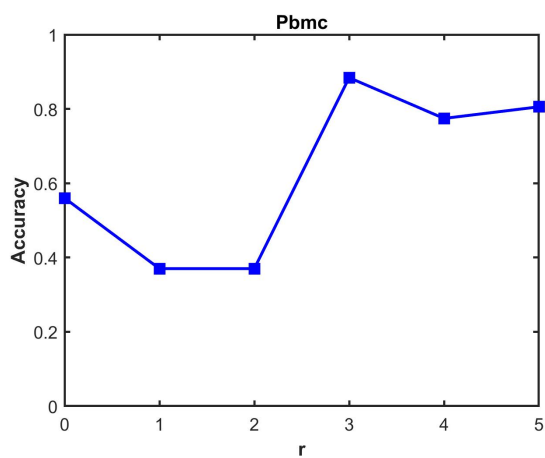

(a)

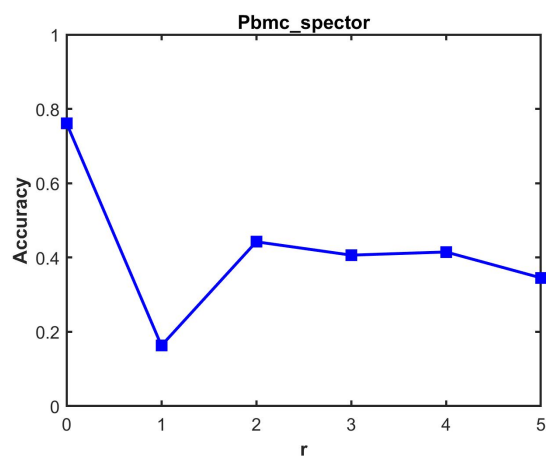

(b)

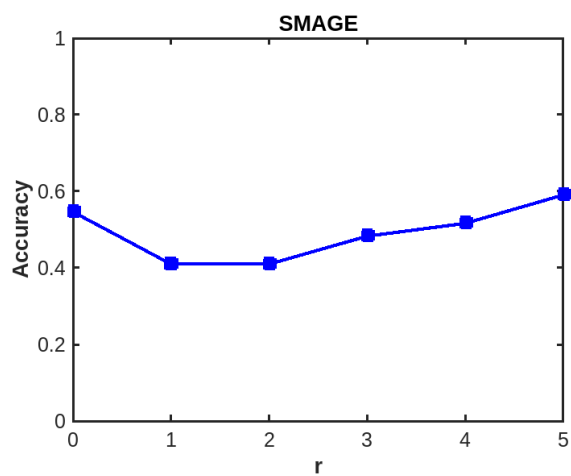

(c)

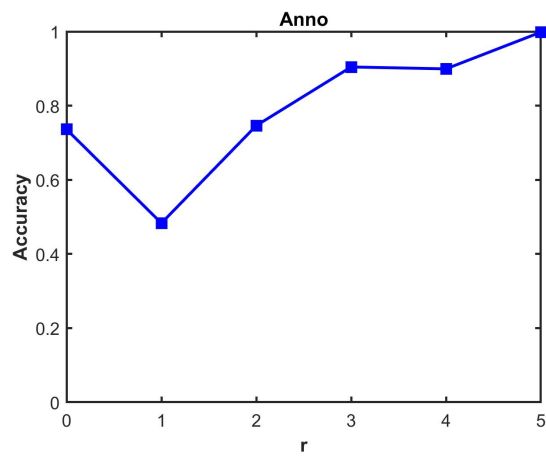

(d)

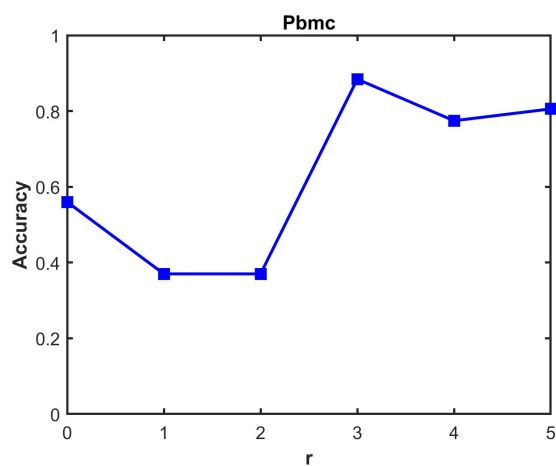

(e)

Line graph with ACC and the parameter  $\gamma$  in (a)-Pbmc, (b)-Spector (c)-SMAGE (d)-Anno (e)-10X\_10K

Supplement: S6 Fig — (PDF) [file pcbi.1013375.s006.pdf]
