# Supplementary material for: PLNMFG: Pseudo-label guided non-negative matrix factorization model with graph constraint for single-cell multi-omics data clustering
Source: PLoS Comput Biol. 2025 Aug 18;21(8):e1013375. doi: 10.1371/journal.pcbi.1013375 (PMC12416850; doi:10.1371/journal.pcbi.1013375)
Supplement: S3 Fig — (a)-BMNC; (b)-10X; (c)-Pbmc; (d)-Anno; (e)-Spector. Each UMAP shows visualization plots of two original omics in the first two panels, and the third panel display visualization plots after processing with PLNMFG. (PDF) [file pcbi.1013375.s003.pdf]

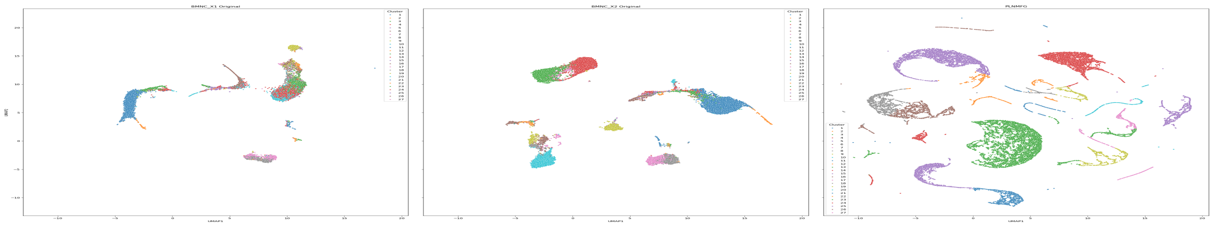

(a)

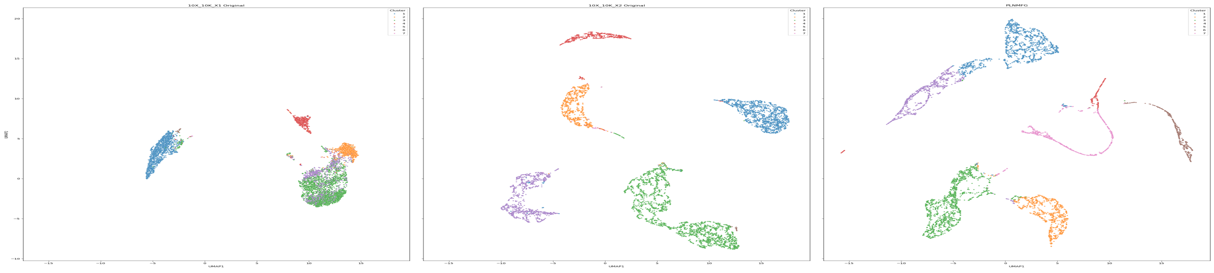

(b)

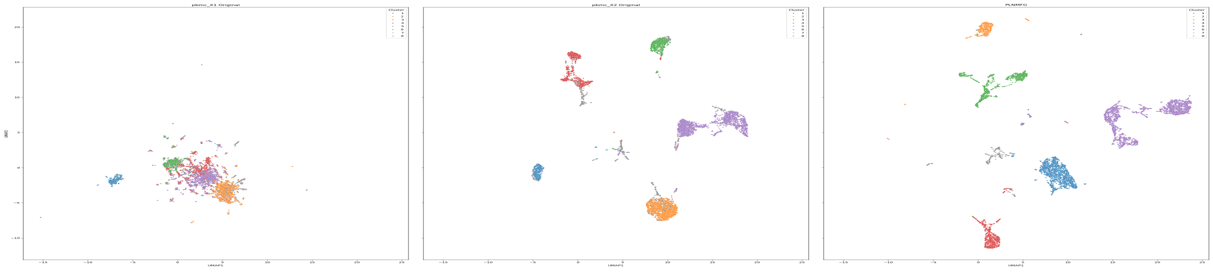

(c)

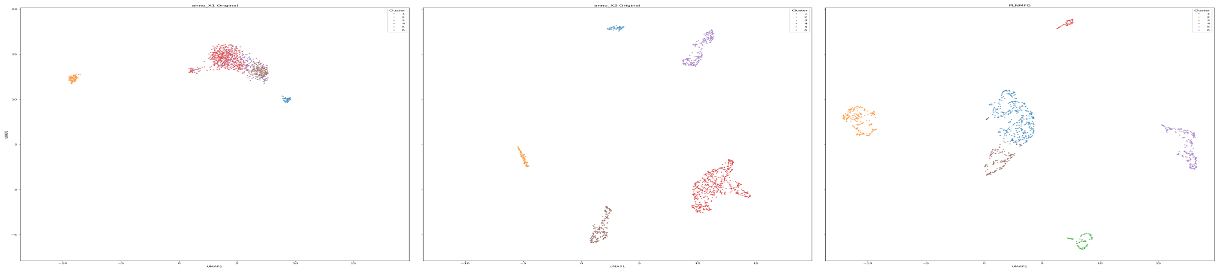

(d)

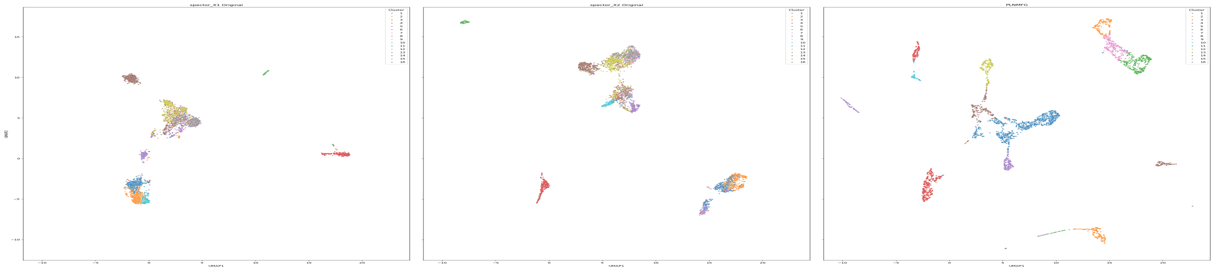

(e)

UMAP visualization plots. The corresponding images and datasets are as follows: (a)-BMNC; (b)-10X_10K. (c)-Pbmc; (d)-Anno; (e)-Spector. Each UMAP shows visualization plots of two original omics in the first two panels, and the third panel display visualization plots after processing with PLNMFG.
