# Supplementary material for: PLNMFG: Pseudo-label guided non-negative matrix factorization model with graph constraint for single-cell multi-omics data clustering
Source: PLoS Comput Biol. 2025 Aug 18;21(8):e1013375. doi: 10.1371/journal.pcbi.1013375 (PMC12416850; doi:10.1371/journal.pcbi.1013375)
Supplement: S7 Fig — (PDF) [file pcbi.1013375.s007.pdf]

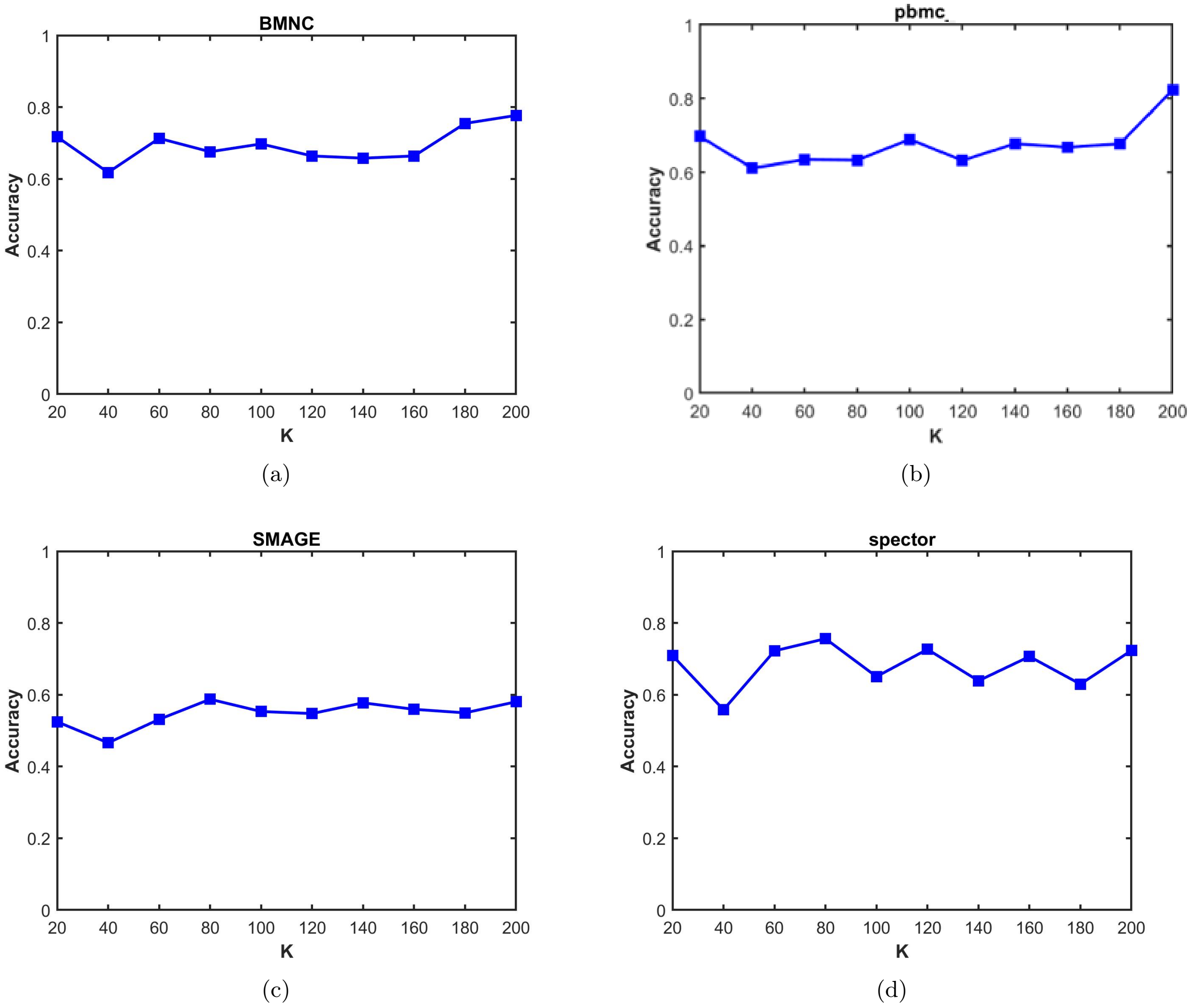

(a) (b) (c) (d)

Line graph shows the relationship between clustering accuracy and the number of clusters (K) for PLNMFG on four different datasets.
