# Supplementary material for: PLNMFG: Pseudo-label guided non-negative matrix factorization model with graph constraint for single-cell multi-omics data clustering
Source: PLoS Comput Biol. 2025 Aug 18;21(8):e1013375. doi: 10.1371/journal.pcbi.1013375 (PMC12416850; doi:10.1371/journal.pcbi.1013375)
Supplement: S1 Text — (PDF) [file pcbi.1013375.s008.pdf]

Parameters

The model consists of six parameters: K, δ, β, ε, η and μ_j. The parameter K represents the latent variables used for dimensionality reduction, and its value is generally selected as an integer within the range of 20 to 200. The parameters δ and β are set with values chosen from the set $\{10^{-4}, 10^{-3}, 10^{-2}, 10^{-1}, 10^{0}\}$. The parameter ε is used to balance the strength of the regularization term and is selected from values such as $10^{-2}, 10^{-1}, \ldots, 10^{3}$. The specific values of K, δ, β and ε can be determined through grid search.

We selected the optimal parameter value η based on cross-validation clustering results.

When

$$(U^i V - V)_{jk} - (S^i)_{jk} > \eta\mu_j$$

we set

$$(S^i)_{jk} = (U^i V - V)_{jk} - (X^i)_{jk} - \eta\mu_j.$$

Otherwise, we consider it as a true "0". By adjusting η, we obtain a threshold to distinguish between biological zeros and non-biological zeros. Different datasets may require different values for η, The optimal value of η for each dataset is determined through grid search.

The parameter μ_j depends on the sequencing depth of the j-th cell. It is defined as follows:

$$\mu_j = \frac{\sum_{j=1}^{p} X_{jk}^i}{median\left\{\sum_{j=1}^{p} X_{jk}^i, \forall j\right\}}.$$

When the sum of the values, $\sum_{j=1}^{p} X_{jk}^i$, is equal to the median of all such sums across cells $\left\{\sum_{j=1}^{p} X_{jk}^i, \forall j\right\}$, $\mu_j = 1$. As the sequencing depth increases, μ_j also increases, reflecting the fact that cells with deeper sequencing generally have fewer "dropout" events.
