# Supplementary material for: PLNMFG: Pseudo-label guided non-negative matrix factorization model with graph constraint for single-cell multi-omics data clustering
Source: PLoS Comput Biol. 2025 Aug 18;21(8):e1013375. doi: 10.1371/journal.pcbi.1013375 (PMC12416850; doi:10.1371/journal.pcbi.1013375)
Supplement: S2 Text — (PDF) [file pcbi.1013375.s009.pdf]

Iteration Process

The objective function is divided into seven manageable subproblems, which are solved iteratively. In each iteration, all variables are fixed except for one, and we update each variable alternately.

(1) Update $\mathbf{U}^i$.

Let the partial derivative of $\mathcal{F}$ with respect to $\mathbf{U}^i$ equal 0, one can obtain

$$\frac{\partial \mathcal{F}}{\partial \mathbf{U}^i} = 2(\alpha_i)^\gamma \mathbf{U}^i \mathbf{V}\mathbf{V}^T - 2(\alpha_i)^\gamma (\mathbf{X}^i + \mathbf{S}^i)\mathbf{V}^T = 0. \tag{1}$$

Then we obtain the closed form solution of $\mathbf{U}^i$:

$$\mathbf{U}^i = \mathbf{V}^T(\mathbf{X}^i + \mathbf{S}^i)(\mathbf{V}\mathbf{V}^T)^{-1} \tag{2}$$

(2) Update $\mathbf{Q}^i$.

Let the partial derivative of $\mathcal{F}$ with respect to Q^i equal 0, we obtain

$$\frac{\partial \mathcal{F}}{\partial \mathbf{Q}^i} = 2(\alpha_i)^\gamma \delta \mathbf{Q}^i \mathbf{V}\mathbf{V}^T - 2(\alpha_i)^\gamma \delta \mathbf{Y}^i \mathbf{V}^T = 0 \tag{3}$$

Then, the closed-form solution of $\mathbf{Q}^i$:

$$\mathbf{Q}^i = \mathbf{Y}^i \mathbf{V}^T (\mathbf{V}\mathbf{V}^T)^{-1} \tag{4}$$

(3) Update $\mathbf{V}$.

Let the partial derivative of $\mathcal{F}$ with respect to $\mathbf{V}$ equal 0, we can obtain

$$\begin{aligned}\frac{\partial \mathcal{F}}{\partial \mathbf{V}} = &\sum_{i=1}^{v} \left[2(\alpha_i)^\gamma \mathbf{U}^{i^T}\mathbf{U}^i + 2(\alpha_i)^\gamma \delta \mathbf{Q}^{i^T}\mathbf{Q}^i\right]\mathbf{V} + 2\beta\mathbf{V} \\ &- 2\beta\mathbf{C}\mathbf{G} \quad - \sum_{i=1}^{v}\left[2(\alpha_i)^\gamma \mathbf{U}^{i^T}(\mathbf{X}^i + \mathbf{S}^i) + 2(\alpha_i)^\gamma \delta \mathbf{Q}^{i^T}\mathbf{Y}^i\right]\end{aligned} \tag{5}$$

Then, the closed-form solution of $\mathbf{V}$ is obtained as follows:

$$\begin{aligned}\mathbf{V} = &\left\{\sum_{i=1}^{v}\left[(\alpha_i)^\gamma \mathbf{U}^{i^T}\mathbf{U}^i + (\alpha_i)^\gamma \delta \mathbf{Q}^{i^T}\mathbf{Q}^i\right] + \beta\mathbf{V}\right\}^{-1} \\ &\times \left\{\sum_{i=1}^{v}\left[(\alpha_i)^\gamma \mathbf{U}^{i^T}(\mathbf{X}^i + \mathbf{S}^i) + (\alpha_i)^\gamma \delta \mathbf{Q}^{i^T}\mathbf{Y}^i\right] + \beta\mathbf{C}\mathbf{G}\right\}\end{aligned} \tag{6}$$

(4) Update $\mathbf{C}$.

Let the partial derivative of $\mathcal{F}$ with respect to $\mathbf{C}$ equal 0, we can obtain

$$\frac{\partial \mathcal{F}}{\partial \mathbf{C}} = 2\beta\mathbf{C}\mathbf{G}\mathbf{G}^T - 2\beta\mathbf{V}\mathbf{G}^T \tag{7}$$

Then, the closed-form solution of $\mathbf{C}$ is obtained as follows:

$$\mathbf{C} = \mathbf{V}\mathbf{G}^T(\mathbf{G}\mathbf{G}^T)^{-1} \tag{8}$$

(5) Update $\mathbf{G}$.

With all variables but $\mathbf{G}$ fixed, we rewrite $\mathcal{F}$ as follows:

$$\frac{\partial \mathcal{F}}{\partial \mathbf{G}} = \beta(-2\mathbf{C}^T\mathbf{V} + 2\mathbf{C}^T\mathbf{C}\mathbf{G}) + 2\varepsilon\mathbf{G}\mathbf{L} \tag{9}$$

Where $L = (D - A)$, then we can obtain the closed-form of $\mathbf{G}$:

$$\mathbf{G} = \mathbf{G}\frac{\beta\mathbf{C}^T\mathbf{V} + \varepsilon\mathbf{G}\mathbf{A}}{\partial\beta\mathbf{C}^T\mathbf{C}\mathbf{G} + \varepsilon\mathbf{G}\mathbf{D}} \tag{10}$$

(6) Update α_i

With all variables but α_i fixed, we rewrite $\mathcal{F}$ as follows:

$$\begin{aligned} &\min \mathcal{F}(\alpha_i) = \sum_{i=1}^{v}(\alpha_i)^\gamma h_i \\ &\text{s.t.} \quad \sum_{i=1}^{v}\alpha_i = 1, \alpha_i > 0. \end{aligned} \tag{11}$$

Here

$$h_i = \|\mathbf{X}^i + \mathbf{S}^i - \mathbf{U}^i\mathbf{V}\|_F^2 + \eta\sum_{j=1}^{n} u_j\|\mathbf{S}_j^i\|_1 + \delta\|\mathbf{Y}^i - \mathbf{Q}^i\mathbf{V}\|_F^2.$$

We can solve the above equation by introducing the Lagrange multiplier method, and Formula (11) becomes:

$$\min F(\alpha_i, \eta) = \sum_{i=1}^{v}(\alpha_i)^\gamma h_i - \eta\left(\sum_{i=1}^{v}\alpha_i - 1\right). \tag{12}$$

Let the partial derivative of $\mathcal{F}$ with respect to α_i equal 0, we can obtain

$$\alpha_i = \left(\frac{\eta}{r h_i}\right)^{\frac{1}{r-1}} \tag{13}$$

Substitute α_i in Eq. (13) into the constraint $\sum_{i=1}^{v}\alpha_i = 1$, we can obtain

$$\alpha_i = \frac{(h_i)^{\frac{1}{1-\gamma}}}{\sum_{i=1}^{v}(h_i)^{\frac{1}{1-\gamma}}} \tag{14}$$

(7) Update $\mathbf{S}^i$.

For the update of the imputation matrix $\mathbf{S}^i$, we apply a Softmax function to compute values for the elements within the set, while assigning zeros to elements outside the set.

We employ the SoftMax function to compute values for elements within the set Ω while set elements outside the set to zero for updating the imputation matrix $\mathbf{S}^i$.

$$\begin{aligned} &\mathbf{S}_{j,k}^i = (soft(\eta), u_j(\mathbf{U}^i\mathbf{V} - \mathbf{X}^i)_{j,k})_+, \quad (j,k) \in \Omega \\ &\mathbf{S}_{j,k} = 0, \quad (j,k) \in \Omega^c. \end{aligned} \tag{15}$$

Here

$$soft(x_i) := \operatorname{sgn}(x^i)(|x^i| - y)_+, \quad (x^i)_+ = \max(x^i, 0)$$

Through the seven iterative steps, we alternately update $\mathbf{U}^i$, $\mathbf{Q}^i$, $\mathbf{V}$, $\mathbf{U}^i$, $\mathbf{G}^i$, α_i and $\mathbf{S}^i$, repeating the process until the objective function converges or the maximum number of iterations is reached. The algorithm for PLNMFG is shown in Table 1

Algorithm 1: Optimize PLNMFG model by alternatingly updating
**Input:** Feature matrices of v views $\{\mathbf{X}^1, \mathbf{X}^2, \ldots, \mathbf{X}^v\}$, the expected number of clusters c, the expected number of latent factors k, parameters γ, θ, β and ε.<br>**Output:** Cluster indicator matrix $\mathbf{G}$, cluster centroid matrix $\mathbf{C}$.<br>**Procedure:**<br>1. Performing k-means on feature matrix of each view to obtain pseudo-label matrix $\mathbf{Y}^i$.<br>2. Initialize $\mathbf{V}^i$ and $\mathbf{C}$ by random matrices.<br>3. Initialize $\alpha^i = \frac{1}{v}$.<br>repeat<br>4.1 Update $\mathbf{U}^i$ by Eq. (2).<br>4.2 Update $\mathbf{Q}^i$ by Eq. (4).<br>4.3 Update $\mathbf{G}$ by Eq. (10).<br>4.4 Update $\mathbf{C}$ by Eq. (8).<br>4.5 Update $\mathbf{V}$ by Eq. (6).<br>4.6 Update α^i by Eq. (14).<br>4.7 Update $\mathbf{S}^i$ by Eq. (15).<br>until converges or reaches the maximum number of iterations.

Table 1. The algorithm for PLNMFG optimization.
