# Supplementary material for: PLNMFG: Pseudo-label guided non-negative matrix factorization model with graph constraint for single-cell multi-omics data clustering
Source: PLoS Comput Biol. 2025 Aug 18;21(8):e1013375. doi: 10.1371/journal.pcbi.1013375 (PMC12416850; doi:10.1371/journal.pcbi.1013375)
Supplement: S3 Text — (PDF) [file pcbi.1013375.s010.pdf]

Data Pre-processing

Before the experiment, we preprocessed the input single-cell multi-omics data matrix $\mathbf{X}^i$ with the following steps:

1. Delete genes that are not expressed on all cells.
2. Filtering out genes that are only present in a small number of cells.
3. Log transformation and normalization.
