# Supplementary material for: PLNMFG: Pseudo-label guided non-negative matrix factorization model with graph constraint for single-cell multi-omics data clustering
Source: PLoS Comput Biol. 2025 Aug 18;21(8):e1013375. doi: 10.1371/journal.pcbi.1013375 (PMC12416850; doi:10.1371/journal.pcbi.1013375)
Supplement: S4 Text — (PDF) [file pcbi.1013375.s011.pdf]

Performance Evaluation

Similar to most clustering methods, we select four commonly used metrics, namely NMI, AMI, ARI, and ACC, as the evaluation criteria.

1 Normalized Mutual Information (NMI)

Normalized mutual information (NMI) is the most widely used measurement for cell types. Specifically, given the standard cluster labels $\mathcal{C}^*$ and obtained cluster labels $\mathcal{C}$, we construct a confusion matrix H whose rows and columns correspond to the cells in $\mathcal{C}^*$ and $\mathcal{C}$, respectively. Element h_{ij} is the number of vertices overlapped by the i-th real and j-th obtained cluster. The NMI is defined as follows:

$$\text{NMI} = \frac{-2\sum_{i=1}^{|\mathcal{C}^*|}\sum_{j=1}^{|\mathcal{C}|} h_{ij}\log\frac{h_{ij}H}{h_{i.}h_{.j}}}{\sum_{j=1}^{|\mathcal{C}^*|} h_{i.}\log\frac{h_i}{H} + \sum_{i=1}^{|\mathcal{C}|} h_{.j}\log\frac{h_j}{H}}$$

where $|\mathcal{C}^*|$ is the number of clusters in $\mathcal{C}^*$ and $h_i.$ is the sum of the i-th row of H.

2 Adjusted Mutual Information (AMI)

The AMI is defined as follows:

$$AMI\left(\mathcal{C}^*, \mathcal{C}\right) = \frac{MI\left(\mathcal{C}^*, \mathcal{C}\right) - E\left\{MI\left(\mathcal{C}^*, \mathcal{C}\right)\right\}}{\max\left(K\left(\mathcal{C}^*\right), K\left(\mathcal{C}\right) - E\left\{MI\left(\mathcal{C}^*, \mathcal{C}\right)\right\}\right)}$$

and

$$MI\left(\mathcal{C}^*, \mathcal{C}\right) = \sum_{i=1}^{|\mathcal{C}^*|}\sum_{i=1}^{|\mathcal{C}|} p\left(i, j\right)\log\frac{p\left(i, j\right)}{p\left(i\right)p\left(j\right)}$$

Where

$$p(i,j) = \frac{|\mathcal{C}_i^* \cap \mathcal{C}_j|}{m}, \quad p(i) = \frac{|\mathcal{C}_i^*|}{m}, \quad p(j) = \frac{|\mathcal{C}_j|}{m}$$

$$K(\mathcal{C}^*) = -\sum_{i=1}^{|\mathcal{C}^*|} p(i)\log p(i), \quad K(\mathcal{C}) = -\sum_{j=1}^{|\mathcal{C}|} p(j)\log p(j)$$

3 Adjusted rand index (ARI)

Performance is measured by the similarity between the estimated cluster labels $\mathcal{C}$ and the true cluster labels $\mathcal{C}^*$ using the ARI

$$\text{ARI} = \frac{\sum_{e,t}\binom{m_{et}}{2} - \frac{\sum_e\binom{m_e}{2}\sum_t\binom{m_t}{2}}{\binom{m}{2}}}{\frac{1}{2}\left(\sum_e\binom{m_e}{2} + \sum_t\binom{m_t}{2}\right) - \frac{\sum_e\binom{m_e}{2}\sum_t\binom{m_t}{2}}{\binom{m}{2}}}$$

where m is the total number of single cells. Here m_e and m_t are the number of single cells in estimated cluster e and in true cluster t, respectively. And m_{et} is the number of single cells shared by estimated cluster e and true cluster t. ARI ranges from 0 to 1, where 1 means the estimated cluster is the same as the true cluster, while 0 means the two are completely different.

4 Clustering Accuracy (ACC)

Let t be the threshold, and $P(t)$ be the set of predicted ontology, and T be the ontology in the single cell dataset. For the i-th single cell, the true positives (TP) and false positives (FP) are defined as follows:

$$TP_i = \sum_{h \in H} I\left(f \in P_i\left(t\right) \wedge f \in T_i\right)$$

$$FP_i = \sum_{h \in H} I\left(f \in P_i\left(t\right) \wedge f \notin T_i\right)$$

where h is an ontology, H denotes the set of all functions, and $I(x)$ is indicator function with value 1 if x is true, 0 otherwise. The accuracy is defined as follows:

$$\text{Accuracy} = \frac{\sum_i TP_i}{\sum_i TP_i + \sum_i FP_i}.$$
