# Supplementary material for: PLNMFG: Pseudo-label guided non-negative matrix factorization model with graph constraint for single-cell multi-omics data clustering
Source: PLoS Comput Biol. 2025 Aug 18;21(8):e1013375. doi: 10.1371/journal.pcbi.1013375 (PMC12416850; doi:10.1371/journal.pcbi.1013375)
Supplement: S5 Text — (PDF) [file pcbi.1013375.s012.pdf]

Convergence Proof

1 Theoretical Analysis of Algorithms

In the algorithm, $\mathbf{U}^i$, $\mathbf{Q}^i$, $\mathbf{V}$, $\mathbf{U}^i$ and $\mathbf{G}^i$ are all updated in elemental units. The stopping criterion of the algorithm can be the maximum number of iterations or the minimum change in the value of the objective function.

Next, we will theoretically prove that Algorithm 1 is convergent and it converges to a limit point. In addition, this limit point satisfies the KKT condition, which is necessary for the local minimum.

Theorem 1 Theoretically Algorithm 1 monotonically decreases the objective function and eventually converges to a limit point.

Proof: In order to prove Theorem 1, we first need to define the upper bound auxiliary function.

Definition 1: If the function $g(h, h')$ is the upper bound auxiliary function of the function $f(h)$, then it satisfies the following conditions:

$$g(h, h') \geq f(h), \quad g(h, h) = f(h). \tag{1}$$

Based on Lemma 1, Theorem 1 can be proved by showing that the upper bound auxiliary functions with respect to $\mathbf{U}^i$, $\mathbf{Q}^i$, $\mathbf{V}$, $\mathbf{C}$, and $\mathbf{G}$ can be minimized individually by proving five update rules (2)(4)(6)(8)(10), as well as by proving that the objective function has a lower bound.

Lemma 1: If g is an upper bound auxiliary function of $f(h)$, then $f(h)$ is non-increasing under the update rule

$$h^{t+1} = \underset{h}{\operatorname{argmin}} g(h, h^t) \tag{2}$$

Proof: $f(h^{t+1}) \leq g(h^{t+1}, h^t) \leq g(h^t, h^t) = f(h^t)$

We first provide the upper bound auxiliary function for $\mathbf{U}^i$ when $\mathbf{Q}^i$, $\mathbf{V}$, $\mathbf{C}$, and $\mathbf{G}$ are fixed, we then prove that (2) optimizes this upper bound auxiliary function.

2 Upper Bound Auxiliary Function for U^i:

The objective function (1) is written in terms of U^i as:

$$O_{U^i} = (\alpha_i)^\gamma \left\| X^i + S^i - U^i V \right\|_F^2 \tag{3}$$

where $\|\cdot\|_F$ is the Frobenius Form of Calculate matrix.

The result of taking the first-order derivative of each element in equation (18) is:

$$F'_{ab} = \frac{\partial O_{U^i}}{\partial U^i_{ab}} = (2(\alpha_i)^\gamma \mathbf{U}^i \mathbf{V}\mathbf{V}^T - 2(\alpha_i)^\gamma (\mathbf{X}^i + \mathbf{S}^i)\mathbf{V}^T)_{ab} \tag{4}$$

The second derivative of O_{U^i} with respect to U^i_{ab} is:

$$F''_{ab} = \frac{\partial^2 O_{U^i}}{\partial U^i_{ab} \partial U^i_{ab}} = 2(\alpha_i)^\gamma (VV^T)_{bb} \tag{5}$$

The higher-order derivative of O_{U^i} with respect to U^i_{ab} is

$$F'''_{ab} = F''''_{ab} = \cdots = 0. \tag{6}$$

Based on the definition of the Taylor expansion, one can rewrite (18) in its Taylor expansion form at the point ${U^i}^t_{ab}$:

$$\begin{aligned} O_{U^i} &= F_{ab} + F'_{ab}(u - {U^i}^t_{ab}) + \frac{F''_{ab}}{2}(u - {U^i}^t_{ab})^2 \\ &= F_{ab} + F'_{ab}(u - {U^i}^t_{ab}) + (\alpha_i)^\gamma (VV^T)_{bb}(u - {U^i}^t_{ab})^2. \end{aligned} \tag{7}$$

Definition 2: A function can be represented as an infinite sum of terms calculated from the derivative of the function at a point:

$$f(y) = \sum_{n=0}^{\infty} \frac{f^{(n)}(a)}{n!}(y-a)^n \tag{8}$$

The expression $f^{(n)}(a)$ represents the nth derivative of the function $f(y)$ at the point a, and $n!$ is the factorial of n. This type of expansion is called the Taylor series expansion.

The upper bound auxiliary function adopted in this paper is defined as (18):

$$G(u, {U^i}^t_{ab}) = F_{ab} + F'_{ab}(u - {U^i}^t_{ab}) + \frac{(\alpha_i)^\gamma (\mathbf{U}^i \mathbf{V}\mathbf{V}^T)_{ab}}{{U^i}^t_{ab}}\left(u - {U^i}^t_{ab}\right)^2. \tag{9}$$

In the following, we prove that $G(u, {U^i}^t_{ab})$ is an upper bounding auxiliary function of (18).

Firstly, it is obvious that when $u = {U^i}^t_{ab}$, $F_{ab} = G(u, {U^i}^t_{ab})$.

Next, we need to prove that $F_{ab} \le G(u, {U^i}^t_{ab})$, which is equivalent to proving:

$$\frac{(\alpha_i)^\gamma (\mathbf{U}^i \mathbf{V}\mathbf{V}^T)_{ab}}{{U^i}^t_{ab}}\left(u - {U^i}^t_{ab}\right)^2 \ge (\alpha_i)^\gamma (VV^T)_{bb}(u - {U^i}^t_{ab})^2. \tag{10}$$

It is straightforward to obtain

$$\begin{aligned} \frac{(\mathbf{U}^i \mathbf{V}\mathbf{V}^T)_{ab}}{{U^i}^t_{ab}} &= \frac{\sum_{k=1}^{K} U^i_{ak} \times (\mathbf{V}\mathbf{V}^T)_{kb}}{{U^i}^t_{ab}} \\ &\ge \frac{U^i_{ab} \times (\mathbf{V}\mathbf{V}^T)_{bb}}{{U^i}^t_{ab}} = (\mathbf{V}\mathbf{V}^T)_{bb}. \end{aligned} \tag{11}$$

We can then conclude that (25) holds, indicating that (24) is an upper bounding auxiliary function of (24). Since (24) is a quadratic convex function, its minimum value can be obtained when:

$$\begin{aligned} {U^i}^{t+1}_{ab} &= {U^i}^t_{ab} - \frac{(2(\alpha_i)^\gamma \mathbf{U}^i \mathbf{V}\mathbf{V}^T - 2(\alpha_i)^\gamma (\mathbf{X}^i + \mathbf{S}^i)\mathbf{V}^T)_{ab}}{2 \times \frac{(\alpha_i)^\gamma (\mathbf{U}^i \mathbf{V}\mathbf{V}^T)_{ab}}{{U^i}^t_{ab}}} \\ &= U^i_{ab} \frac{\left[\left(X^i + S^i\right) V^T\right]_{ab}}{\left[U^i V V^T\right]_{ab}} \end{aligned} \tag{12}$$

and this is exactly the update rule of $\mathbf{V}$ in (6).

In the same manner, we can design upper bound auxiliary functions for $\mathbf{Q}_i$, $\mathbf{V}$, $\mathbf{C}$, $\mathbf{G}$, and prove that their minimum values will be achieved under the update rules (4), (6), (8) and (10). Moreover, it is obvious that the objective function has 0 as its lower bound.

Therefore, we can conclude that the algorithm can converge to a limit point. The necessary condition for a local optimal solution is that the limit point must satisfy the KKT conditions. As shown in Theorem 1, the proposed algorithm converges to a limit point.

Theorem 2: When initialized with a positive matrix, i.e $\mathbf{Q}_i$, $\mathbf{Q}_i$, $\mathbf{V}$, $\mathbf{C}$, $\mathbf{G} > 0$, the algorithm can converge to a limit point that satisfies the KKT conditions.

Proof: The Lagrange function of the objective function is expressed as follows:

$$\begin{aligned} L = \sum_{i=1}^{\nu} (\alpha_i)^{\gamma} \Bigg\{ & \left\| X^i + S^i - U^i V \right\|_F^2 + \eta \sum_{j=1}^{n} u_j \left\| S_j^i \right\|_1 \\ & + \delta \left\| Y^i - Q^i V \right\|_F^2 \Bigg\} + \beta \left\| V - CG \right\|_F^2 + \varepsilon \mathrm{Tr}(GLG^T) \\ & - \sum_{i=1}^{\nu} \left(\mathrm{Tr}(\Phi_{U^i} U^i) + \mathrm{Tr}(\Phi_{Q^i} Q^i) \right) - \mathrm{Tr}(\Phi_V V) - \mathrm{Tr}(\Phi_C C) - \mathrm{Tr}(\Phi_G G) \end{aligned} \tag{13}$$

Where Φ_{U^i}, Φ_{Q_i}, Φ_V, Φ_C, Φ_G are respectively the Lagrange multiplier matrices. Based on the Lagrangian function in (28), the KKT conditions for the objective funciton are summarized as:

$$\begin{cases} U^i \geq 0 \quad i = 1 \cdots v \\ Q^i \geq 0 \quad i = 1 \cdots v \\ V \geq 0 \\ C \geq 0 \\ G \geq 0 \\ 2\mathbf{U}^i \mathbf{V}\mathbf{V}^T - 2(\mathbf{X}^i + \mathbf{S}^i)\mathbf{V}^T - \Phi_{U^i} = 0 \quad i = 1 \cdots v \\ 2\mathbf{Q}^i \mathbf{V}\mathbf{V}^T - 2\mathbf{Y^i}\mathbf{V}^T - \Phi_{Q_i} = 0 \quad i = 1 \cdots v \\ 2\sum_{i=1}^{\nu} \left[(\alpha_i)^{\gamma} {U^i}^T U_i + (\alpha_i)^{\gamma} \delta {Q^i}^T Q_i \right] + 2\beta V \\ -2\sum_{i=1}^{\nu} \left[(\alpha_i)^{\gamma} {U^i}^i (X^i + S^i) + (\alpha_i)^{\gamma} \delta {Q^i}^T Y^i \right] \\ +2\beta CG - \Phi_V = 0 \\ 2\beta CGG^T - 2\beta VG^T - \Phi_C = 0 \\ \beta(-2C^T V + 2C^T CG) + 2\varepsilon GL - \Phi_G = 0 \\ (\Phi_{U^i})_{ab} U_{ab}^i = 0 \quad \forall a, b \quad i = 1 \cdots v \\ (\Phi_{Q_i})_{ab} Q_{ab}^i = 0 \quad \forall a, b \quad i = 1 \cdots v \\ (\Phi_V)_{ab} V_{ab} = 0 \quad \forall a, b \\ (\Phi_C)_{ab} C_{ab} = 0 \quad \forall a, b \\ (\Phi_G)_{ab} G_{ab} = 0 \quad \forall a, b \\ \Phi_{U^i} \geq 0; \Phi_{Q_i} \geq 0; \Phi_V \geq 0; \Phi_C \geq 0; \Phi_G \geq 0 \end{cases} \tag{14}$$

Among them, 0 is the zero matrix. Let $\{(U^i)^t\}_{t=1}^{\infty}$ represent the update sequence, and $\{(U^i)^*\} = \{\lim_{t \to +\infty} (U^i)^t\}$ is the limit point that the algorithm will converge to.

When the algorithm converges, we have $(U^i)_{ab}^* = (U^i)_{ab}^{\infty} = (U^i)_{t+1}^{ab} = (U^i)_t^{ab}$. Therefore, the updating rule for U^i becomes:

$$(U^i)_{ab}^* = (U^i)_{ab}^* \frac{\left[\left(X^i + (S^i)^* \right) V^{*T} \right]_{ab}}{\left[(U^i)^* V^* V^{*T} \right]_{ab}} \tag{15}$$

Therefore, we obtain

$$\left[\left(X^i + (S^i)^*\right) V^{*T}\right]_{ab} = \left[(U^i)^* V^* V^{*T}\right]_{ab}. \tag{16}$$

Since problem has constraints, i.e., $U^i \geq 0$ either $(U^i)^*_{ab} > 0$ or $(U^i)^*_{ab} = 0$.
Case 1: If $U^*_{ab} > 0$ then we have

$$\left[\left(X^i + (S^i)^*\right) V^{*T}\right]_{ab} = \left[(U^i)^* V^* V^{*T}\right]_{ab}. \tag{17}$$

And accordingly $(\Phi_{U^i})_{ab} = 0$, which satisfies the KKT conditions.
Case 2: If $U^*_{ab} = 0$ then we have

$$\begin{aligned}
0 = (\mathbf{U^i})^*_{\mathbf{ab}} &= (\mathbf{U^i})^{\mathbf{0}}_{\mathbf{ab}} \lim_{t\to\infty} \prod_{r=0}^{t} \frac{\left[\left(X^i + (S^i)^r\right) V^{rT}\right]_{ab}}{\left[(U^i)^r V^r V^{rT}\right]_{ab}} \geq 0 \\
\implies \lim_{t\to\infty} \frac{\left[\left(X^i + (S^i)^t\right) V^{tT}\right]_{ab}}{\left[(U^i)^t V^t V^{tT}\right]_{ab}} &= \frac{\left[\left(X^i + (S^i)^*\right) V^{*T}\right]_{ab}}{\left[(U^i)^* V^* V^{*T}\right]_{ab}} \leq 1 \\
\implies (\Phi_{U^i})_{ab} &= \left[(U^i)^* V^* V^{*T}\right]_{ab} - \left[\left(X^i + (S^i)^*\right) V^{*T}\right]_{ab} \geq 0.
\end{aligned} \tag{18}$$

This also satisfies the KKT condition.
Using the same method, we can obtain the relationships that $\mathbf{Q}^i$, $\mathbf{V}$, $\mathbf{C}$, $\mathbf{G}$ and Φ_{Q_i}, Φ_V, Φ_C, Φ_G also satisfy the KKT condition.
